# Leucine‐Dependent SLC7A5–PGAM5 Interaction Promotes Advanced Atherosclerosis Through Hindering Mitochondrial Function of Macrophages

**DOI:** 10.1002/advs.202518359

**Published:** 2025-11-21

**Authors:** Shan Zhong, Xueyu Wang, Qingsong Li, Siqi Wang, Bin Sun, Wenjun Ni, Gengyu Zhou, Fan Wang, Xianwei Xie, Cheng Jin, Gang Xu, Peng Zhao, Xiang Peng, Feiyuan Han, Xiangwen Xi, Yidan Wang, Juan Xu, Yue Wang, Xia Gu, Shuo Li, Jian Zhang, Shuijie Li, Jinwei Tian

**Affiliations:** ^1^ Department of Cardiology The Second Affiliated Hospital of Harbin Medical University Harbin 150001 P. R. China; ^2^ Heilongjiang Provincial Key Laboratory of Panvascular Disease Harbin 150081 P. R. China; ^3^ The Key Laboratory of Myocardial Ischemia Ministry of Education Harbin Medical University Harbin 150001 P. R. China; ^4^ BGI Research Beijing 102601 P. R. China; ^5^ Collaborative Innovation Center for Molecular Imaging of Precision Medicine Shanxi Medical University Taiyuan Shanxi 030001 P. R. China; ^6^ Research Center for Pharmacoinformatics (the State‐Province Key Laboratories of Biomedicine‐Pharmaceutics of China) College of Pharmacy Harbin Medical University Harbin 150081 P. R. China; ^7^ College of Pharmacy Harbin Medical University Harbin 150081 P. R. China; ^8^ College of Bioinformatics Science and Technology Harbin Medical University Harbin Heilongjiang 150081 P. R. China; ^9^ Department of Cardiology the Second Affiliated Hospital of Hainan Medical University Haikou 570311 P. R. China; ^10^ State Key Laboratory of Quality Research in Chinese Medicine and Institute of Chinese Medical Sciences University of Macau Macao P. R. China; ^11^ Cardiovascular Imaging Center The Second Affiliated Hospital of Harbin Medical University Harbin Heilongjiang 150001 P. R. China; ^12^ School of Pharmacy East China University of Science and Technology Shanghai 200237 P. R. China; ^13^ Department of Biopharmaceutical Sciences College of Pharmacy Harbin Medical University Harbin 150081 P. R. China; ^14^ Heilongjiang Province Key Laboratory of Research on Molecular Targeted Anti‐Tumor Drugs Harbin 150000; ^15^ State Key Laboratory of Frigid Zone Cardiovascular Diseases (SKLFZCD) Harbin Medical University Harbin 150081 P. R. China; ^16^ Department of Geriatrics The Second Affiliated Hospital of Harbin Medical University Harbin 150001 P. R. China

**Keywords:** advanced atherosclerosis, leucine, macrophages, mitochondria, PGAM5, SLC7A5

## Abstract

The residual risks of advanced atherosclerosis remain substantial despite current preventive strategies and pharmacotherapy. Circulating branched‐chain amino acids are biomarkers of cardiovascular disease risk. However, the mechanism of leucine in atherosclerosis progression remains unclear. Leucine transporter‐SLC7A5‐mediated leucine intake that promotes advanced atherosclerosis in mice, increasing apoptotic macrophages and lipids accumulation within plaques. Multi‐omics analyses showed that leucine deprivation enhanced macrophage mitochondrial function and increased plaque CD5L^hi^ macrophages, under SLC7A5‐deficiency‐mediated leucine deprivation, these cells exhibited stronger oxidative phosphorylation and lipid metabolism. Mechanistically, leucine deficiency reduced SLC7A5‐PGAM5 binding in macrophages, promoting PGAM5‐NDUFV1 interaction and enhancing mitochondrial function, which attenuates atherosclerosis progression. Collectively, these findings elucidate the function and mechanism of SLC7A5 in *Cd5l* ^hi^ macrophages, highlighting it as a potential therapeutic target. Strategies aimed at improving mitochondrial function also offer a promising approach for advanced atherosclerosis treatment.

## Graphical abstract

Higher plasma leucine is associated with increased risk of new‐onset myocardial infarction. Leucine deprivation alleviates advanced atherosclerosis in mice. Tumor‐induced leucine deprivation reprograms macrophage metabolism and increases CD5L^hi^ macrophages in mouse plaques. Mechanistically, leucine deficiency reduced SLC7A5‐PGAM5 binding in macrophages, promoting PGAM5‐NDUFV1 interaction and enhancing mitochondrial function, which attenuates atherosclerosis progression.

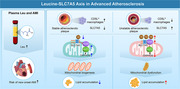

## 1 Introduction

Advanced atherosclerosis (AS) is the leading cause of various vascular diseases, including ischemic heart disease and stroke, and peripheral arterial disease.^[^ ^1^ ^]^ Most recurrent clinical events after contemporary treatment, involving lipid‐ or glucose‐lowering agents, healthy lifestyles, and anti‐thrombotic therapies are associated with AS.^[^ ^2^ ^]^ Recent advances in the human genome, metabolome and clinical studies have enhanced our understanding of AS. Circulating branched‐chain amino acids (BCAAs), including isoleucine, valine and leucine plays crucial roles in AS.^[^ ^3^, ^4^, ^5^ ^]^ BCAAs are risk biomarkers,^[^ ^6^, ^7^ ^]^ however, their mechanisms, especially leucine, in AS progression have not been fully elucidated. As essential amino acids, BCAAs account for 20% of total protein intake.^[^ ^8^ ^]^ Therefore, defining strategies for vascular disease prevention is essential, considering the relationship between essential BCAA intake and metabolic abnormalities leading to AS.

Leucine is the key activator of mTOR signaling in macrophages^[^ ^9^ ^]^ and in tumor cells^[^ ^10^ ^]^ and a substrate for protein synthesis, which is essential for tumor survival and proliferation. Its metabolites also participate in the tricarboxylic acid (TCA) cycle. Macrophages may utilize leucine as an alternative carbon source to glucose and glutamine during inflammatory responses,^[^ ^11^ ^]^ reshaping immune function and cytokine production. Notably, activated pro‐inflammatory macrophages are characterized by reduced oxidative phosphorylation (OXPHOS) and TCA cycle activity and enhanced aerobic glycolysis, resulting in less efficient ATP production.^[^ ^12^ ^]^ Consistently, a deficiency of leucine degradation enzyme leads to leucine accumulation in the aorta and impairs mitochondrial function and broader metabolic pathways in macrophages.^[^ ^13^ ^]^ However, whether AS progression is influenced by the direct inhibition of leucine uptake in macrophages—and by what mechanisms—remains unclear.

Solute carrier family seven member 5/L‐type amino acid transporter 1 (SLC7A5/LAT1), primarily transports leucine into cells.^[^ ^10^ ^]^ Beyond its established roles in tumor growth and proliferation—making it a therapeutic target^[^ ^14^, ^15^ ^]^—SLC7A5 reprograms immune cell metabolism and is crucial for T‑cell development, activation, and differentiation.^[^ ^16^, ^17^ ^]^ Given the central role of macrophage metabolic state in plaque biology, we hypothesized that SLC7A5‑mediated leucine uptake regulates macrophage mitochondrial function and thereby influences AS progression.

In this study, we investigate the role and mechanism of leucine and SLC7A5 in AS progression. An analysis of the UK Biobank (UKB) database revealed elevated plasma leucine levels as a risk factor for new‐onset acute myocardial infarction (AMI). Through multi‐omics analysis of mouse plaques, we identified the foamy macrophage subcluster characterized by high expression of CD5 antigen‐like protein/apoptosis inhibitor of macrophages (CD5L/AIM) and upregulated under leucine deficiency as the primary cluster with enhanced mitochondrial‐related functions. Furthermore, we identified phosphoglycerate mutase 5 (PGAM5), a mitochondrial phosphatase that regulates mitochondrial dynamics and stress signaling,^[^ ^18^, ^19^ ^]^ as a protein that directly binds with SLC7A5 and enhances mitochondrial function through its interaction with NADH: ubiquinone oxidoreductase core subunit V1 (NDUFV1). NDUFV1, a core complex I subunit that is essential for electron transport and OXPHOS; mutations in NDUFV1 lead to various mitochondrial disorder.^[^ ^20^, ^21^, ^22^ ^]^ Taken together, our work elucidates the role of leucine in AS and the underlying molecular mechanisms, providing insights for therapeutic strategies.

## 2 Results

### 2.1 Leucine Deprivation Alleviates Advanced AS Via Reduced Macrophage Apoptosis

**Figure 1:**
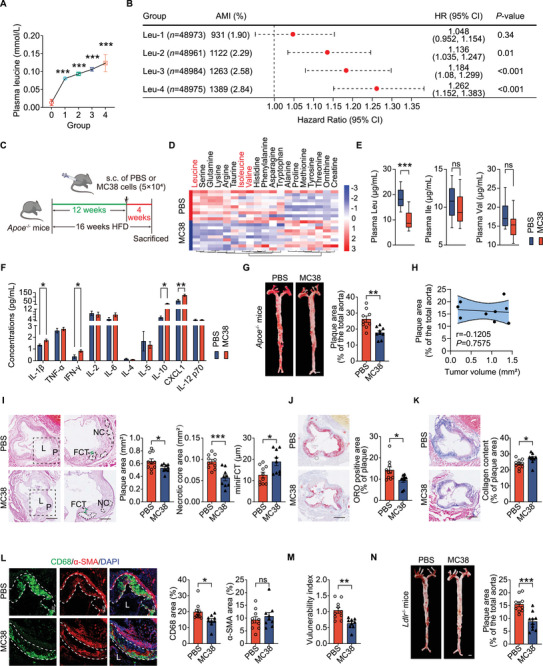
Subcutaneous MC38 tumors reduce plasma leucine and atherosclerotic lesion size in mice. A) Average plasma leucine level in each group (*n* = 48961‐48984/group). B) Association between plasma leucine levels and AMI risk. C) Schematic of the AS mouse model comorbid with subcutaneous colon cancer (MC38). D) Heatmap of plasma amino acids levels of *Apoe* ^−/−^ mice with (MC38) or without tumors (PBS), fed with an HFD for 16 weeks (*n =*10 mice/group). E) Box diagrams showing plasma BCAAs levels in MC38‐ and PBS‐treated *Apoe* ^−/−^ mice fed an HFD for 16 weeks (*n =*10 mice/group). F) Luminex analysis of plasma cytokines in indicated mice (*n* = 10 mice/group). G) *En face* Oil red O (ORO) staining (left) and its quantification (right) in indicated mice fed an HFD for 16 weeks (*n =*9‐10/group). Scale: 1 mm. H) Correlation analyses of total aortic plaque area and tumor volume in MC38 group (*n =*9). I‐K, H&E staining (I), ORO staining (J), and Masson staining (K) of aortic sinus plaque (*n =*9‐10/group). L) lumen. P) plaque. Scale: 500 µm and 200 µm(high magnification). L) Immunofluorescence staining of CD68 (macrophages) and α‐SMA (smooth muscle cells) (*n =*9‐10/group). Scale: 100 µm. M) Plaque vulnerability index. Vulnerability index was calculated as lipid deposit and macrophages/collagen fibers and SMCs (*n =*9‐10/group). N, *En face* ORO staining of aortas from *Ldlr* ^−/−^ mice bearing subcutaneous MC38 tumors, implanted at week 12 during a 16‐week HFD (*n =*10‐11/group). Scale: 1 mm. Data are mean ± SEM. Each data point represents an independent biological sample. ns, not significant, *P<0.05, **P<0.01, ***P<0.001, one‐way ANOVA (A), Cox‐regression analysis (B), unpaired Student's *t*‐test (E–G and I–N), and Pearson's correlation test (H). HR, Hazard Ratio. CI, Confidence interval.

Cox regression analysis using the UKB data revealed a significant association between higher plasma leucine levels, after adjusting for age and sex (Table S1, Supporting Information), and an increased risk of new‐onset AMI during follow‐up. Compared with the Leu‐0 group, the hazard ratios (HRs) of the Leu‐2, Leu‐3, and Leu‐4 groups were 1.136 (95% CI 1.035–1.247; *P* = 0.01), 1.184 (95% CI 1.08–1.299; *P*<0.001) and 1.262 (95% CI 1.152–1.383; *P*<0.001), respectively (**Figure** **1**A,B). We then assessed the relationship between plasma BCAA levels and comorbid cancers in human cohorts. Following propensity score matching (PSM) for age, sex and body mass index (BMI), paired Student's *t*‐test analysis of 8226 participant pairs from the UKB database revealed significantly lower plasma leucine (0.096±0.024 vs 0.097±0.024) and isoleucine (0.047±0.015 versus 0.048±0.015) in individuals with cancer than in those without (Figure S1A and Table S2, Supporting Information). Since leucine is essential for tumor growth and is consumed in large quantities by tumors,^[^ ^23^, ^24^ ^]^ we established a co‐morbid animal model in which tumor implantation during AS progression induced systemic leucine deprivation, enabling investigation of leucine's role in AS under complex pathological conditions. Using liquid chromatography‐high‐resolution mass spectrometry (LC‐MS) metabolomics on ten paired plasma samples from mice subcutaneously injected with the MC38 colorectal cancer cells (MC38 group) or phosphate buffered saline (PBS) (PBS group) during a 16‐week high‐fat diet (HFD) feeding regimen (Figure 1C), we found that leucine was the most significantly reduced metabolite (Figure 1D,E), supporting tumor‐induced systemic leucine depletion.

Although tumors also broadly altered systemic inflammation—plasma Luminex profiling showed increased pro‑inflammatory IL‑1β, IFN‑γ, and CXCL1, along with elevated IL‑10, an anti‑inflammatory cytokine (Figure 1F)—the MC38 group nonetheless exhibited reduced aortic plaque area compared to controls (Figure 1G). Aortic plaque area did not correlate with tumor volume in MC38‐bearing *Apoe* ^−/−^ mice (Figure 1H), likely because tumor sizes were strictly constrained to a narrow range (maximum diameter<15 mm in any dimension). MC38‐bearing *Apoe* ^−/−^ mice showed reduced plaque vulnerability versus PBS controls, with smaller necrotic core area, greater minimum fibrous cap thickness (mini FCT) (Figure 1I), lower lipid content (Figure 1J), increased collagen content (Figure 1K) and decreased macrophage (CD68^+^ area) content (Figure 1L). Smooth muscle cells (SMCs) (α‐SMA^+^ area) (Figure 1L) within plaques remained unchanged. Consequently, the plaque vulnerability index^[^ ^25^ ^]^ was significantly lower (Figure 1M). Similar results were obtained from *Ldlr* ^−/−^ mice that received MC38 cells, with reductions in aortic plaque area (Figure 1N). Body weight (Figure S1B, Supporting Information) and food intake (Figure S1C, Supporting Information) did not differ between MC38‐ and PBS‐treated *Apoe* ^−/−^ mice. Similarly, plasma lipids—including total cholesterol (TC), triglycerides (TGs), high‐density lipoprotein (HDL) and low‐density lipoprotein (LDL)—were comparable between MC38‑ and PBS‑treated groups in both *Apoe* ^−/−^ and *Ldlr* ^−/−^ mice (Figure S1D,E, Supporting Information). These findings suggest that MC38 tumors affect advanced atherosclerotic lesions, might be associated with plasma leucine levels and are independent of plasma lipids.

**Figure 2:**
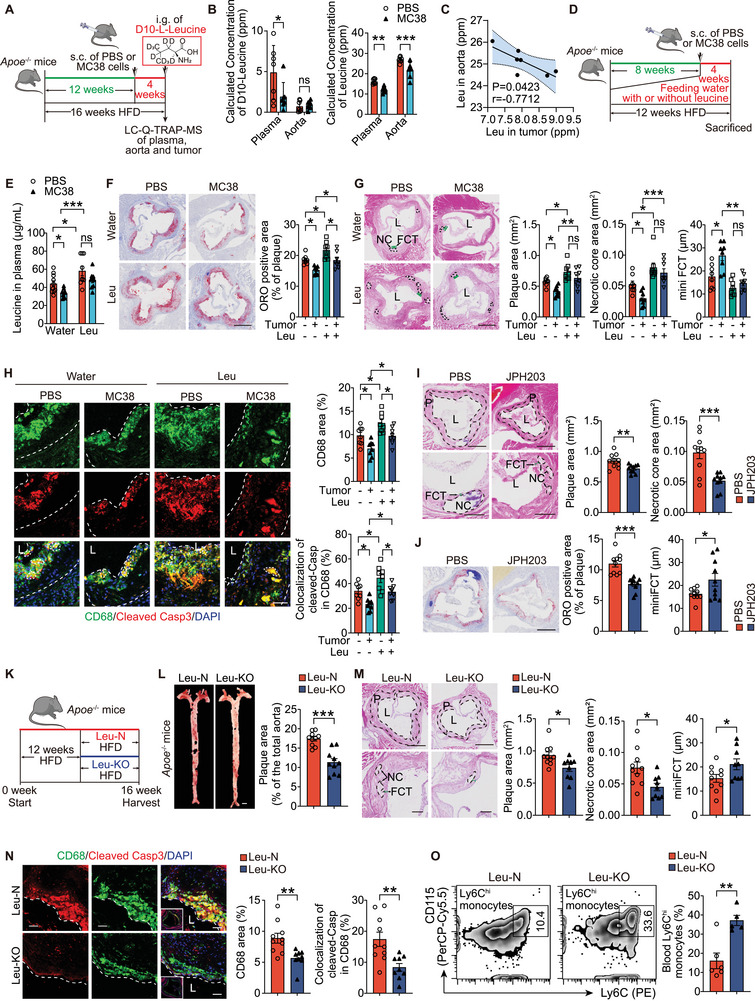
Leucine deprivation alleviates advanced AS via reduced macrophage apoptosis. A) Schematic of the experimental design including LC–Q‐TRAP‐MS analysis of plasma, aortic, and tumor leucine levels from atherosclerotic mice with (MC38) or without (PBS) tumors, which received D10‐labeled leucine. *Apoe* ^−/−^ mice fed a HFD for 12 weeks received subcutaneous PBS or MC38 cells. D10‐labeled L‐leucine was administered at the end of 4 weeks after tumor cell treatment, and samples were collected within 1.5 h (*n =*7/group). B,C) D10‐L‐leucine (left) and leucine (right) levels in plasma and aortas (B) of the indicated mice and correlation of endogenous leucine levels in aortas and in tumors (C). D) Schematic of the experimental design for administering excess leucine to *Apoe* ^−/−^ mice treated with PBS or MC38. E) Plasma leucine levels in *Apoe* ^−/−^ mice that received water with or without excess leucine and treated with PBS or MC38. *n* = 9‐10 mice/group. F,G) ORO staining of aortic sinus (F), and H&E (G) staining of aortic sinus from the indicated mice (*n =*8/group). Scale: 500 µm. H) Immunofluorescent of CD68‐positive areas and apoptotic macrophages (CD68^+^Cleaved Casp3^+^) in mouse aortic sinus (*n =*8/group). L) lumen. Scale: 200 µm and 50 µm (high magnification). I,J) H&E staining (I) and ORO (J) of aortic sinus from *Apoe* ^−/−^ mice fed an HFD for 12 weeks and subsequent daily intraperitoneally injections of JPH203 (6.7 mgkg^−1^) or PBS as a control for 4 weeks (*n =*10/group). Scale: 500 and 200 µm (high magnification). K) Schematic of the experimental design. *Apoe* ^−/−^ mice were fed either a leucine‐restricted high‐fat diet (0% leucine; Leu‐KO) or a normal high‐fat diet (1.6% leucine; Leu‐N) for 4 weeks. L,M) *En face* Oil red O (ORO) staining (L) and H&E staining of aortic sinus (M). N) Immunofluorescent CD68^+^ and CD68^+^Cleaved Caspase3^+^ macrophages in aortic sinus (*n =*9‐10/group). L) lumen. Scale: 50 µm. O, Quantification of Ly6C^hi^ blood monocytes in indicated groups (*n =*5‐6/group). Data are mean ± SEM. ns, not significant, *P<0.05, **P<0.01, ***P<0.001, two‐way ANOVA with Dunnett correction (B), Pearson's correlation test (C), unpaired Student's *t*‐test (E, I, J, and L‐O) and one‐way ANOVA (F‐H).

To detect leucine distribution in vivo in the presence of tumors, mice in the MC38 and PBS groups were gavaged with deuterium‐10 (D10)‐labeled leucine, and targeted metabolite detection was performed (**Figure** **2**A). Using LC‐quadrupole‐trap‐MS, we detected D10‐leucine in the aorta, plasma and tumor tissue at 1.5 h post‐gavage. The MC38 group exhibited significantly lower plasma D10‐leucine levels than the PBS group, although the levels in the aortas did not differ between both groups within this short period post gavage (Figure 2B). However, the MC38 group exhibited significantly lower leucine levels in both plasma and aorta than in the PBS group (Figure 2B). Leucine levels in the aortas and tumors in the MC38 group were significantly inversely correlated (Figure 2C), indicating that tumors may deprive the aortas of leucine and possibly the serum.

To confirm the association between the leucine deprivation by tumor and AS development, MC38 tumor‐bearing *Apoe^−/−^* mice were fed an HFD with drinking water supplemented with or without excess leucine (Figure 2D). Leucine supplementation increased plasma leucine levels that were reduced by tumors (Figure 2E). In *Apoe^−/−^* mice that received or did not receive tumor implantation, excess leucine in the drinking water significantly increased lipid contents (Figure 2F) and necrotic core area (Figure 2G). Supplementation with excess leucine abolished the tumor's effect on plasma leucine levels (Figure 2E), plaque areas, necrotic core areas or fibrous cap thickness (Figure 2G) in *Apoe^−/−^* mice. Tumor implantation reduced plaque lipid content (Figure 2F) and macrophages, including apoptotic macrophages (cleaved‐caspase3/CD68 co‐staining, Figure 2H), whereas excess leucine increased these macrophages (Figure 2H). However, neither leucine supplementation nor tumor implantation altered macrophage proliferation within the plaque (Figure S1F, Supporting Information) or plasma lipid levels (Figure S1G, Supporting Information). These findings underscore the critical role of MC38 tumor‐induced leucine deprivation in atherogenesis.

RNA‐sequencing of the aortic tissues from normal diet (NOR group) and AS mice fed an HFD for 16 weeks (AS group)^[^ ^26^, ^27^ ^]^ revealed impaired OXPHOS and BCAA degradation during AS progression (Figure S2A, Supporting Information), which may cause excess leucine deposition within the plaque. To test whether direct leucine deprivation affects AS development, *Apoe^−/−^* mice were intraperitoneally injected daily with the selective SLC7A5 inhibitor JPH203 (Nanvuranlat), which inhibits leucine uptake and cell proliferation,^[^ ^14^, ^28^ ^]^ or PBS as a control for 4 weeks during the 16‐week HFD regimen to verify whether blocking the excessive uptake of plasma leucine into cells alleviates AS. JPH203 alleviated AS, evidenced by reduced atherosclerotic area, necrotic core area (Figure 2I), lipid content (Figure 2J), and plaque macrophage content (Figure S2B, Supporting Information), as well as an increase in fibrous cap thickness (Figure 2I). However, SMCs (Figure S2B, Supporting Information) did not differ. *Apoe* ^−/−^ mice subjected to dietary leucine deprivation (0% leucine; Leu‐KO, Figure 2K) showed significantly reduced plaque area (Figure 2L‐M) and fewer apoptotic macrophages within plaques (Figure 2N) compared with Leu‐N controls. Although the blood monocytes counts remained unchanged (Figure S2C,D, Supporting Information), the Leu‐KO group had higher activated monocytes compared to the Leu‐N group (Figure 2O). Transwell assays further demonstrated enhanced CCR2‐dependent migratory capacity of blood monocytes in Leu‐KO mice (Figure S2E, Supporting Information). Therefore, we concluded that leucine deprivation alleviates advanced AS in mice, primarily by reducing plaque macrophages counts and macrophage apoptosis.

### 2.2 CD5L is Increased in Metabolically Reprogrammed Macrophages Within Atherosclerotic Lesions

**Figure 3:**
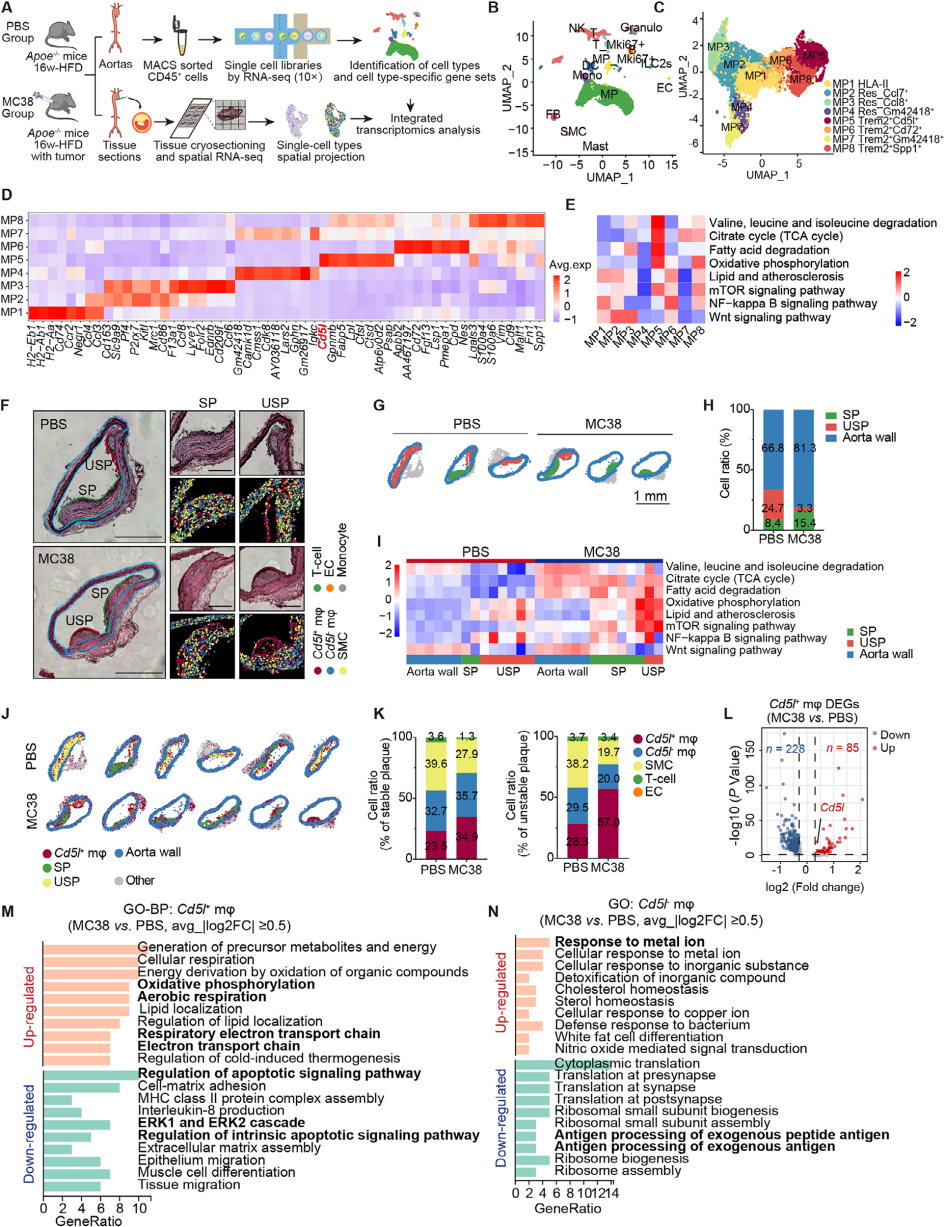
CD5L/AIM upregulation in metabolically reprogrammed plaque macrophages. A) Schematic of the experimental design and analysis. Mouse aortas (depicted in Figure 1B and *n =*25) processed for: single‐cell RNA‐sequencing (scRNA‐seq, CD45^+^ sorting, 10×, clustering); spatial transcriptomics (ST, aortic root cryosections, spot‐based localization); integrated mapping of cell populations and subpopulations. were digested into a single‐cell suspension. B,C) UMAP plot of all cells colored based on the 14 cell types (24 132 cells from 50 mice) (B) and 8 macrophage subclusters (13 680 cells from 50 mice) (C) identified from scRNA‐seq of mouse aortas. D) Heatmap of top eight DEGs in each macrophage subcluster (MP1‐MP8). E) Heatmap of scaled module scores of genes annotated to selected KEGG pathways in different macrophage subclusters (MP1‐MP8). F) H&E staining and graph‐based clustering of *Cd5l* ^+^ and *Cd5l* ^−^ macrophage, SMCs, T‐cells, endothelial cells, and monocytes of the origin of the ascending aortas from *Apoe* ^−/−^ mice treated with PBS or MC38. SP: stable plaque, USP: unstable plaque. Scale: 500 µm and 200 µm (high magnification). G) Annotations of stable plaque (green), unstable plaque (red), aortic wall (blue), and perivascular fat and connective tissue (gray) in the aortas. Scale: 1 mm. H) Stacked histogram of the composition of three regions (SP, USP, and aortic wall) of each group of mice. I) Heatmap of scaled add‐module scores for selected KEGG pathways in three regions (blue, aorta wall; yellow, USP; green, SP). J) Location distribution of *Cd5l* ^+^ macrophage in aortic plaques and the aortic wall. K) Stacked histogram of main cell type compositions in stable (left) and unstable (right) plaques from both groups of mice. L) Volcano plot analysis of DEGs in *Cd5l* ^+^ macrophage (log2 FC≥0.25, *P*<0.05) from the aortic roots from the PBS and MC38 group. M,N) GO (BP) pathway for DEGs (MC38 vs PBS group, |log2 FC|≥0.5) in *Cd5l* ^+^ (M) and *Cd5l* ^−^ (N) macrophage subclusters.

To assess macrophage changes in atherosclerotic lesions under tumor‐mediated leucine deprivation, we performed single‐cell RNA‐sequencing (scRNA‐seq) on sorted CD45^+^ leukocytes from the aortas of HFD‐fed *Apoe^−/−^* mice treated with MC38 or PBS. Integration with spatial transcriptomic data allowed localization of cell clusters within plaques (**Figure** **3**A). Using scRNA‐seq, we identified 14 cell types, including a small number of SMCs, endothelial cells (ECs), and fibroblasts (Figure 3B, Figure S3A, Supporting Information). *Mki67* (a proliferation‐associated gene) expression in plaque macrophages did not differ between the MC38 and PBS groups; however monocytes in the MC38 group exhibited elevated *Mki67* (Figure S3B, Supporting Information). Macrophages were classified into eight subsets based on classical populations and metabolic status (Figure 3C,D). *Trem2* ^+^ *Cd5l* ^+^ macrophages (MP5) exhibited the highest anti‐inflammatory score (Figure S3C, Supporting Information) and increased BCAAs degradation, TCA cycle, and OXPHOS activities compared with the other subclusters (Figure 3E). Pseudotime trajectory analysis indicated *Trem2* ^+^ macrophages (MP5‐MP8) were strongly enriched in cell fate 1, which was associated with lipolysis, apoptosis, OXPHOS, and broader metabolic pathways (Figure S3D–H, Supporting Information). In contrast, HLA‐II macrophages (MP1) exhibited the highest pro‐inflammatory score (Figure S3C, Supporting Information), while resident‐like macrophages (MP2‐MP3) were enriched in cell fate 2 (Figure S3D–G, Supporting Information). Transcription factor regulation activity prediction revealed that *Gabpb1* and *Nfe2l1*, which regulate mitochondrial transcription factor A (TFAM), may be involved in transcription in MP5 cells (Figure S3I, Supporting Information).

We mapped gene expression and cellular composition in plaques of PBS and MC38 groups by spatial transcriptomics of aortic roots, integrating single‐cell data and annotating histological features with H&E staining (Figure 3F). Two blinded cardiologists delineated four regions in each section: aortic wall, stable plaque (SP), unstable plaque (USP) and perivascular adipose connective tissue (other) (Figure 3G). Primarily, USP is characterized by a thin fibrous cap, significant calcifications, or a necrotic lipid core without nuclei.^[^ ^29^, ^30^ ^]^ The proportion of cells in the USP area was significantly lower in the MC38 group (Figure 3H). In MC38 lesions, BCAA degradation, TCA cycle, and fatty acid degradation activities were higher than in PBS controls; compared with normal aortic walls, plaques showed higher OXPHOS but lower BCAA degradation, indicating impairment of this pathway during AS progression; overall damage, including the aortic wall, SP and USP, was less severe in the MC38 group than in the PBS group (Figure 3I; Figure S4A, Supporting Information).

Among all MP5 marker genes, *Cd5l* exhibited the highest average fold change in expression (Figure S4B, Supporting Information). CD5L/AIM—a key regulator of lipid synthesis—is primarily expressed by macrophages. It reduces lipid droplet size in adipocytes.^[^ ^31^ ^]^ In contrast to other marker genes of *Trem2* ^+^ macrophages, *CD5L* expression was lower in human atherosclerotic plaques than in normal human aortas (GSE40231) (*n* = 40 per group) (Figure S4C, Supporting Information). Cell annotation revealed a relatively greater percentage of *Cd5l* ^+^ macrophages in the plaque area of the aortic roots from the MC38 group than in the control group (Figure 3J,K), in both SP and USP regions (Figure 3K). The percentage of *Cd5l* ^−^ macrophages in the SP regions remained comparable between the MC38 and PBS groups, however, it was much lower in the USP regions of the MC38 group than in the PBS group (Figure 3K). In *Cd5l* ^+^ macrophages, the average *Cd5l* expression in the MC38 group was also significantly higher than that in the PBS group (Figure 3L), and *Cd5l* ^hi^ cells were mostly located in the fibrous cap regions (Figure S4D, Supporting Information). Gene Ontology (GO) enrichment analysis enhanced OXPHOS and electron transport chain functionality but weakened apoptosis and inflammatory pathway activation capacity in *Cd5l* ^+^ macrophages from the MC38 group than in those from the control group (Figure 3M). Additionally, *Cd5l* ^−^ macrophages from the MC38 group exhibited a stronger stimuli response and a weaker response to antigen presentation than those from the PBS group (Figure 3N). Therefore, *Cd5l* ^hi^ macrophages were identified as critical cell clusters that increase in plaques with enhanced mitochondrial‐related functions.

### 2.3 Leucine Deficiency Enhances Oxidative Phosphorylation of Macrophages, Reducing Lipid Accumulation in Atherosclerotic Lesions

**Figure 4:**
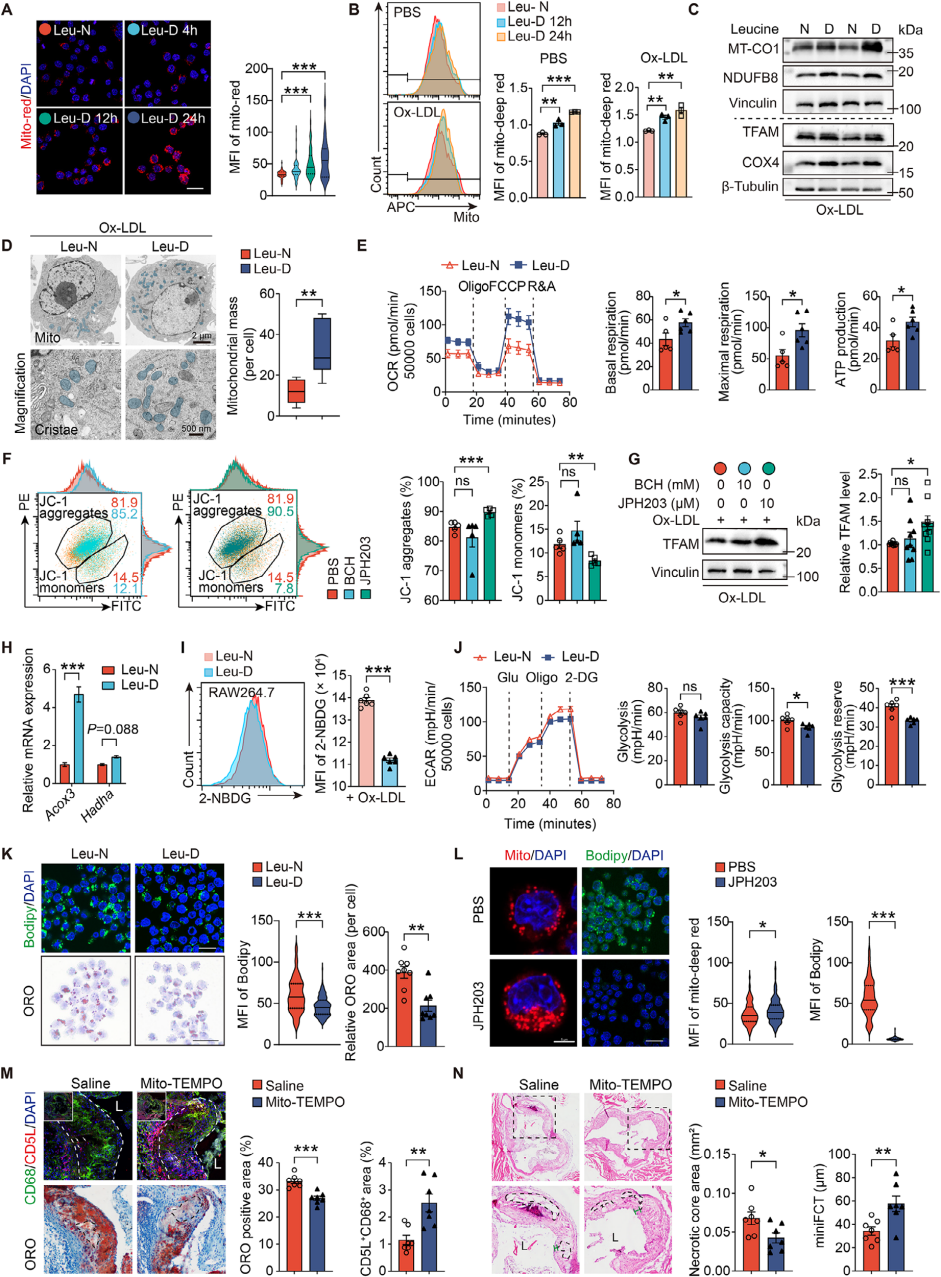
Leucine deficiency enhances oxidative phosphorylation of macrophages, reducing lipid accumulation in atherosclerotic lesions. A) Immunofluorescence staining of MitoTracker‐red in RAW264.7 cells cultured in leucine‐normal (Leu‐N) and leucine deficiency (Leu‐D) media (*n*≥100 cells/group). Scale: 20 µm. B) Flow cytometry analysis of MitoTracker‐deep red in RAW264.7 cells treated with PBS or ox‐LDL (50 µg/mL) and in Leu‐N and Leu‐D media at different times (*n =*3/group). C) Immunoblot analysis of OXPHOS‐related protein and TFAM in RAW264.7 cells after 24‐h ox‐LDL treatment with or without 12‐h leucine deficiency (*n*≥7/group). D) Representative transmission electron microscopy images of mitochondria (left top), mitochondrial cristae (left bottom), and mitochondrial mass (right) in RAW264.7 cells after 24‐h ox‐LDL (50 µgmL^−1^) treatment with or without 12‐h leucine deficiency (*n* = 8/group). Scale, 1 µm and 200 nm (high magnification). E) Seahorse XF96 mitochondrial respiration in ox‑LDL–treated RAW264.7 macrophages under leucine deficiency (Leu‑D) or normal leucine (Leu‑N), assessing basal and maximal oxygen consumption rate (OCR) and ATP production after sequential injection of oligomycin 1.5 µM FCCP 1.5 µM and rotenone/antimycin A 0.5 µM (*n =*5‐6/group). F) Mitochondrial membrane potential depolarization of RAW264.7 cells stimulated for 18 h with PBS, BCH (10 mM), or JPH203 (10 µM) during 24‐h ox‐LDL (50 µgmL^−1^) treatment, assessed using flow cytometry with mitochondrial membrane potential assay kit JC‐1 (*n =*5/group). G) Immunoblot analysis of TFAM in RAW264.7 cells under indicated treatment (*n =*9/group). H) Quantitative RT‐PCR analysis of fatty acid oxidation (FAO)‐related genes *Acox3* and *Hadha* from RAW264.7 cells treated with ox‑LDL for 24 h with or without 12 h leucine deficiency (*n* = 3/group). I) Histogram and quantification of 2‐NBDG signal in RAW264.7 cells under indicated treatment (*n* = 6). J) ECAR of RAW264.7 cells under indicated treatment (*n* = 6). K) BODIPY^493/503^ (top, *n*≥100 cells/group) or ORO staining (bottom, *n =*8 visual fields per group, each containing over 100 cells) of ox‐LDL‐treated RAW264.7 cells in Leu‐N and Leu‐D media. Scale: top 20 µm, bottom, 50 µm. L) MitoTracker‐red (left and center) and BODIPY^493/503^ (right) staining of RAW264.7 cells after 18‐h treatment with PBS or JPH203 (10 µM) during 24‐h ox‐LDL treatment (*n*≥100 cells/group). Scale: 20 µm and 5 µm (high magnification). M) Immunofluorescent staining of CD68^+^CD5L^+^ cells (top) and ORO staining (bottom) of aortic root plaque in *Apoe* ^−/−^ mice fed an HFD for 16 weeks and received mito‐TEMPO (0.8 mgkg^−1^ body weight) or saline (*n =*7/group). Scale: 50 µm. N) H&E staining of indicated mice (*n =*7/group). Scale: 500 and 200 µm (high magnification). Data are mean ± SEM. ns, not significant, *P<0.05, **P<0.01, ***P<0.001. Kruskal‐Wallis test (A), one‐way ANOVA (B), unpaired Student's *t*‐test (D‐G, I, ORO staining in K, and M‐N), two‐way ANOVA with Dunnett correction (H), Mann‐Whitney *U* test (BODIPY^493/503^ staining in K and L).

Omics analysis revealed that the metabolic status of the macrophages differed under physiological and atherosclerotic conditions (Figure 3I), prompting us to explore whether leucine deficiency could reprogram their metabolism. Leucine deprivation causes a time‐dependent increase in mitochondrial mass in macrophages, as shown by MitoTracker staining of RAW264.7 cells, a mouse macrophage cell line (**Figure** **4**A,B). The mouse bone marrow‐derived macrophages (BMDMs) demonstrated similar results (Figure S4A, Supporting Information). Leucine deficiency increased the mitochondrial transcription factor TFAM, OXPHOS‐related proteins (cytochrome c oxidase subunit 1, MT‐CO1 and NDUFB8) and cytochrome c oxidase subunit 4 (COX4, a mitochondrial marker) levels (Figure 4C; Figure S4B, Supporting Information) in ox‐LDL‐stimulated RAW264.7 cells. It also increased mitochondrial mass and restored cristae as assessed using transmission electron microscopy (Figure 4D). Regarding mitochondrial function, leucine deficiency increased the cellular oxygen consumption rate (OCR) in RAW264.7 cells (Figure 4E). Both JPH203 and 2‐aminobicyclo(2,2,1)heptane‐2‐carboxylic acid (BCH; a non‐selective leucine transporter inhibitor) inhibited leucine transport into cells.^[^ ^14^ ^]^ Mitochondrial membrane potential indicates cell health and mitochondrial functional status.^[^ ^32^ ^]^ JPH203, not BCH, increased macrophage mitochondrial membrane potential (JC‐1 aggregates) (Figure 4F) and TFAM expression (Figure 4G), and reduced apoptotic macrophages (JC‐1 monomers) (Figure 4F) in the presence of ox‐LDL.

Under leucine deprivation, oxLDL‐stimulated RAW264.7 macrophages exhibited increased *Acox3* expression, a key FAO enzyme (Figure 4H), accompanied by reduced glucose uptake (Figure 4I) and lower extracellular acidification rate (ECAR), indicating suppressed glycolysis (Figure 4J). Concordantly, leucine deficiency also reduced RAW264.7 cell intracellular lipid contents (Figure 4K), consistent with a metabolic shift away from glycolysis and toward fatty acid catabolism. Leucine transportation inhibition with JPH203 significantly increased mitochondrial mass and reduced intracellular lipid contents in ox‐LDL‐stimulated RAW264.7 cells (Figure 4L).

Mitochondrial activators play a role in attenuating AS progression.^[^ ^33^, ^34^ ^]^ We explored whether this effect involves metabolic reprogramming of macrophages. Mito‐TEMPO, a reactive oxidative species (ROS) scavenger, reduced lipid content in RAW264.7 cells (Figure S5C, Supporting Information), decreased lesion lipid deposition (Figure 4M) and necrotic core area (Figure 4N), and increased *Cd5l* ^+^ macrophage content (Figure 4M) and fibrous cap thickness (Figure 4N) in the aortic root atherosclerotic plaques of *Apoe* ^−/−^ mice on an HFD for 16 weeks. These findings revealed metabolic reprogramming in the atherosclerotic lesions of mice treated with mitochondrial activators.

### 2.4 Silencing SLC7A5 Mitigates AS by Enhancing the Mitochondrial Function of Macrophages

**Figure 5:**
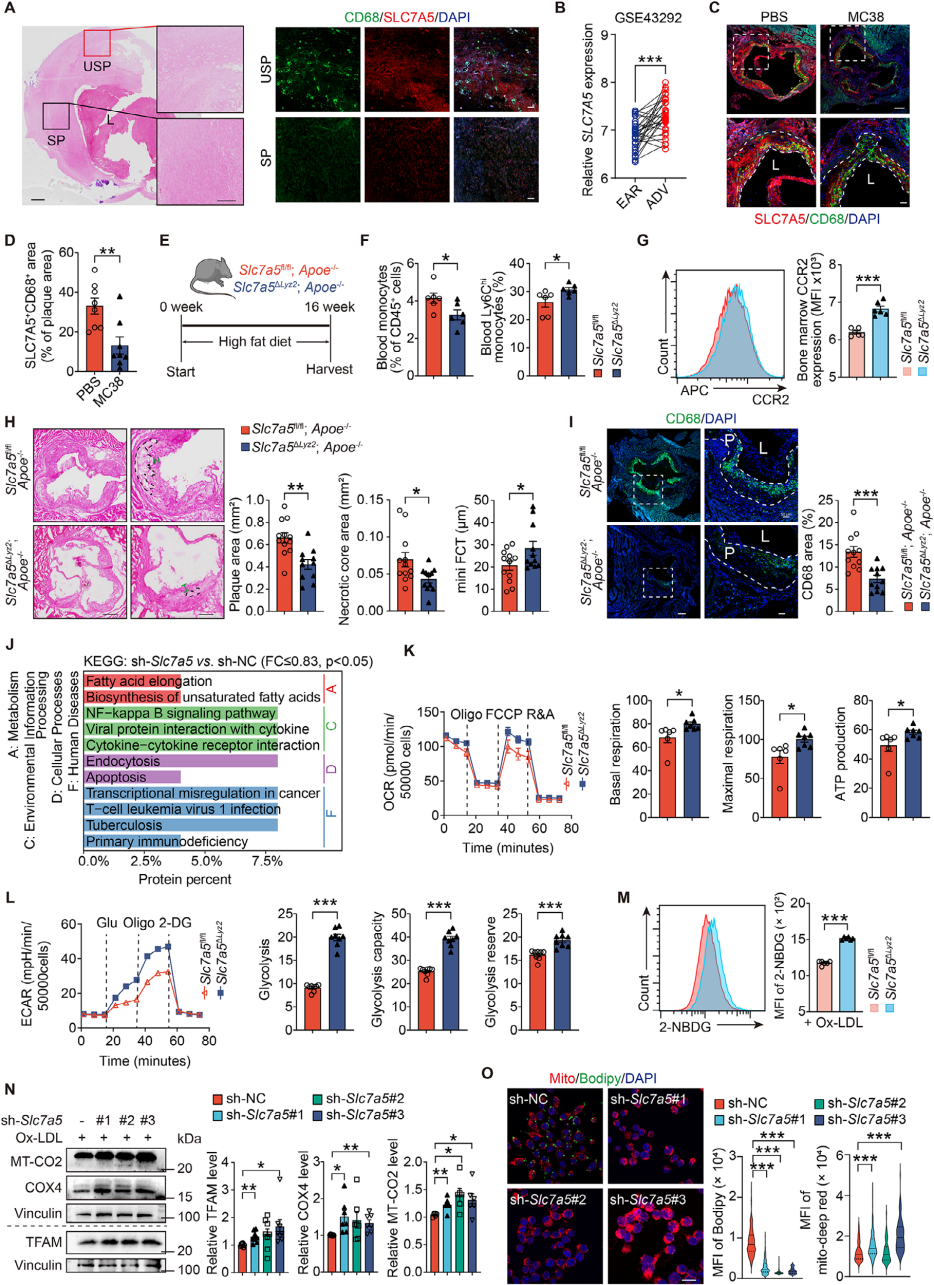
Silencing SLC7A5 mitigates AS by enhancing the mitochondrial function of macrophages. A) H&E (left, scale: 1 mm and 500 µm) and immunofluorescent staining of SLC7A5 and CD68 (right, scale: 50 µm) of human femoral artery atherosclerotic plaques. SP, stable plaque. USP, unstable plaque. L, lumen. B) *SLC7A5* mRNA levels in human advanced carotid atherosclerotic plaques (stages IV and V) compared with paired early plaques (stages I and II) in GSE43292 dataset (*n* = 32/group). C,D) Immunofluorescence staining (C) and quantification of SLC7A5 (red) and CD68 (green) in the aortic sinus of *Apoe* ^−/−^ mice comorbid with MC38 (*n* = 7‐8/group). Scale: 200 and 50 µm (high magnification). E) Schematic of the experimental design of *Slc7a5* ^fl/fl^ *; Apoe* ^−/−^ mice and *Slc7a5* ^fl/fl^ *Lyz2*‐cre; *Apoe* ^−/−^ mice fed an HFD for 16‐weeks (*n* = 11‐12 mice/group). F) Quantification of total (left) and Ly6C^hi^ (right) blood monocytes from *Slc7a5^ΔLyz2^* and *Slc7a5* ^fl/fl^ mice (*n =*6/group) G) Quantification of CCR2 expression on blood monocytes from indicated mice (*n* = 6/group). H,I) H&E (H) and immunofluorescent staining of CD68 (I) of the aortic sinus of indicated mice (*n* = 11‐12 mice/group). Scale in H: 250 and 100 µm (high magnification). Scale in I: 200 and 50 µm (high magnification). J) Top ten enriched KEGG pathways identified for regulated proteins with FC≤0.83, *P*<0.05 in RAW264.7 cells transfected with sh‐*Slc7a5* versus negative control sh‐NC. K,L) OCR (K) and ECAR (L) of BMDMs from the *Slc7a5* ^fl/fl^ mice and *Slc7a5^ΔLyz2^* mice following 24‐h ox‐LDL (50 ug/mL) treatment (*n* = 6 or 8). M) Histogram and quantification of 2‐NBDG signal in RAW264.7 cells under indicated treatment (*n* = 6). N) Immunoblot of TFAM, COX4, and MT‐CO2 in RAW264.7 cells with three *Slc7a5*‐knockdown sequences (#1, #2, #3) following 24‐h ox‐LDL (50 µgml^−1^) treatment (*n*≥6/group). O) Immunofluorescence of MitoTracker‐deep red and BODIPY^493/503^ in RAW264.7 cells with indicated treatment (*n*≥100 cells/group). Scale: 20 µm. Data are presented as mean ± SEM. *P<0.05, **P<0.01, ***P<0.001, paired Student's *t*‐test (B), unpaired Student's *t*‐test (D, F‐I and K‐N), and Kruskal‐Wallis test (O).

Given that the selective SLC7A5 inhibitor JPH203 enhances mitochondrial function and mitigates AS, we investigated the role of SLC7A5 in AS progression. Immunofluorescent staining detected a much higher *SLC7A5* expression in unstable regions of human femoral artery plaques than in stable regions (**Figure** **5**A). The GEO database GSE43293 also demonstrated similar results. *SLC7A5* expression was increased in the core area of human carotid plaques than in the stable area (Figure 5B). Consistent with the hypothesis that high *SLC7A5* expression in lesion macrophages is associated with AS progression, the CD68^+^SLC7A5^+^ area in plaques from tumor‐bearing (MC38 cells) *Apoe^−/−^* mice was significantly lower than that in PBS‐treated mice (Figure 5C,D). Furthermore, *SLC7A5* mRNA expression was negatively correlated with *TFAM* but positively correlated with fatty acid binding protein 4 (*FABP4*)—a foamy macrophage marker linked to plaque rupture—in human atherosclerotic plaques^[^ ^35^ ^]^ (Figure S6A, Supporting Information), indicating that elevated SLC7A5 in lesion macrophages is associated with foam cell formation.

To further demonstrate the potential role of SLC7A5 in AS progression, we generated myeloid‐specific *Slc7a5* knockout mice with an *Apoe* ^−/−^ background (*Slc7a5^ΔLyz2^*; *Apoe* ^−/−^) (Figure S6B, Supporting Information), exhibiting significantly lower *Slc7a5* mRNA expression in BMDMs (Figure S6C, Supporting Information). Eight‐week‐old *Slc7a5^ΔLyz2^*; *Apoe* ^−/−^ mice and their littermate (*Slc7a5* ^fl/fl^; *Apoe* ^−/−^) were fed an HFD for 16 weeks (Figure 5E). Body weight (Figure S6D, Supporting Information), liver lipid levels (Figure S6E, Supporting Information), or plasma lipid levels did not significantly differ between the groups (Figure S6F, Supporting Information). Myeloid‐specific deletion of *Slc7a5* had no effect on the number or activation profile of bone marrow monocytes (Figure S6G,H, Supporting Information). However, in the blood, monocyte counts decreased while the proportion of activated subsets increased (Figure 5F), accompanied by elevated CCR2 expression (Figure 5G). These observations suggest that *Slc7a5* deletion increases monocyte migration to tissues. However, Myeloid‐specific SLC7A5 deficiency in *Apoe* ^−/−^ mice significantly reduced aortic roots plaque size, necrotic core area (Figure 5H), lipid content (Figure S6I, Supporting Information), and macrophage content (CD68^+^ area; Figure 5I), while increasing fibrous cap thickness (Figure 5H) and collagen content (Figure S6J, Supporting Information), and lowered plaque vulnerability (Figure S6K, Supporting Information). Notably, expression of efferocytosis‐related genes (*Mertk* and *Tim4*) in peritoneal macrophages remained unchanged for both groups (Figure S6L, Supporting Information). Therefore, myeloid‐specific *Slc7a5* deficiency mitigates AS primarily via direct effects on plaque macrophages, leading to reduced lipid loading.

To directly evaluate *Slc7a5* role in macrophage foam cell formation, we constructed both *Slc7a5*‐knockdown (sh‐*Slc7a5*) and exogenous *Slc7a5*‐FLAG overexpressing in RAW264.7 cells, followed by TMT quantitative proteomic detection and analysis. The knockdown efficiency of SLC7A5 expression in RAW264.7 cells was confirmed using either RT‐PCR (Figure S7A, Supporting Information) or immunoblot analysis (Figure S7B, Supporting Information). KEGG enrichment analysis showed that the proteins downregulated in sh‐*Slc7a5* versus sh‐NC were enriched in fatty acid biosynthesis, transcription factors of inflammation, and apoptosis pathways (Figure 5J). Gene set enrichment analysis (GSEA) revealed decreased expression of proteins involved in OXPHOS and electron transport chain pathways following *Slc7a5* overexpression in RAW264.7 cells (Figure S7C, Supporting Information). Western blotting further confirmed these changes in ox‐LDL‐treated SLC7A5‐overexpressing RAW264.7 cells, showing reduced expression of TFAM, MT‐CO1, and the key rate‐limiting enzyme for β‐oxidation, carnitine palmitoyl transferase 1a (CPT1A) (Figure S7D, Supporting Information). The mRNA levels of FAO (*Acox3, Hadha* and *Hadhb*) and TCA‐related genes (*Idh3a*) were significantly reduced in *Slc7a5*‐overexpressing macrophages (Figure S7E, Supporting Information). Regarding metabolic compensation, unlike leucine deprivation, *Slc7a5^ΔLyz2^* BMDMs showed increased OXPHOS and glycolytic capacities (Figure 5K,L), along with higher glucose uptake (Figure 5M). This metabolic upshift was associated with increased mitochondrial mass (Figure S7F, Supporting Information) and fewer foam cells under ox‐LDL treatment (Figure S7G, Supporting Information). Furthermore, *Slc7a5* knockdown in RAW264.7 cells increased TFAM and OXPHOS‐related protein expression (Figure 5N), enhanced mitochondrial function (Figure S7H, Supporting Information) and membrane potential (JC‐1 aggregates) (Figure S7I, Supporting Information), elevated mitochondrial mass, and reduced lipid content under ox‐LDL stimulation (Figure 5O). Collectively, SLC7A5 impairs mitochondrial metabolism and promotes foam cell formation.

### 2.5 SLC7A5 Directly Binds to PGAM5 in Mitochondria

**Figure 6:**
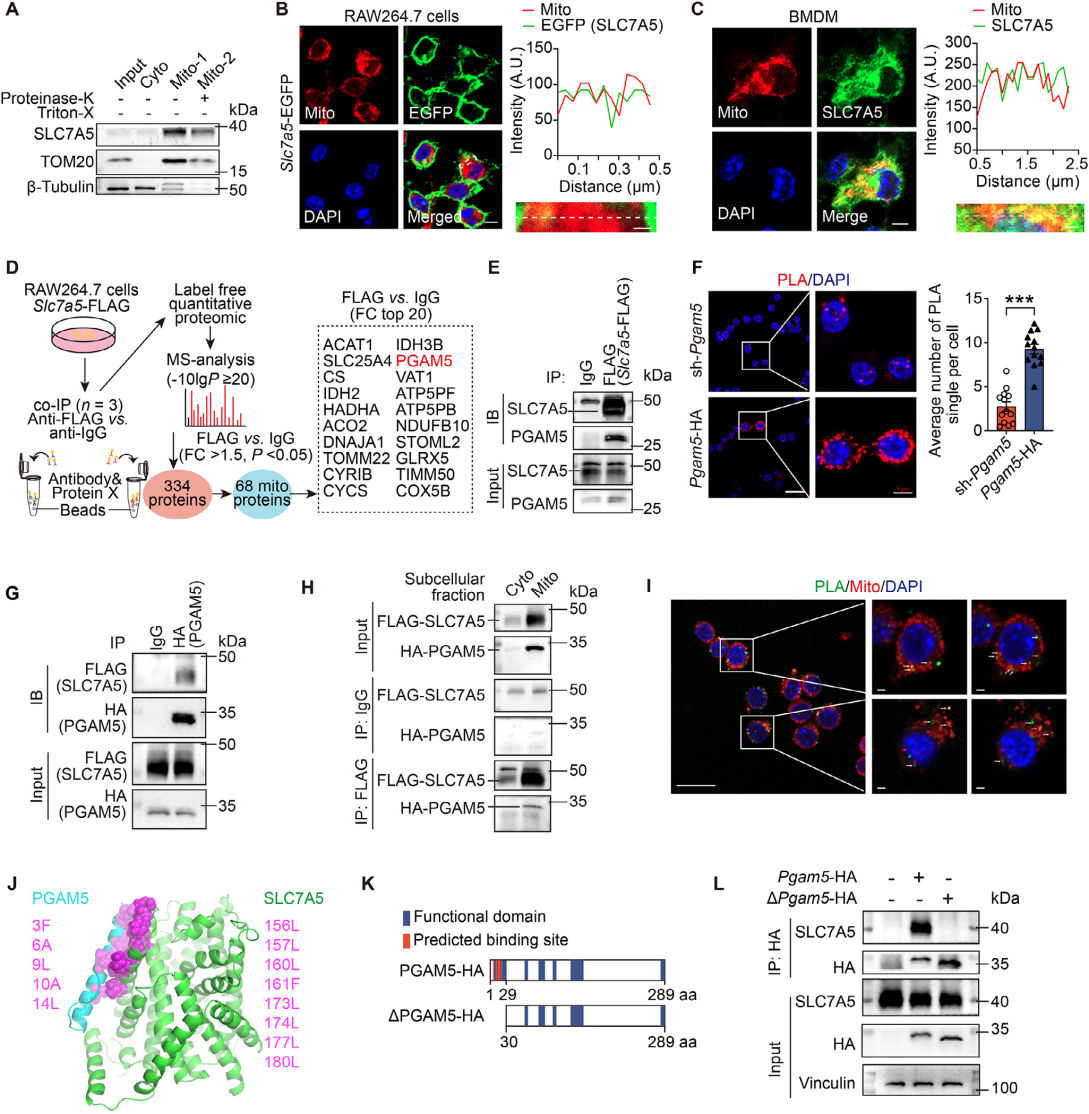
SLC7A5 directly binds to PGAM5 in mitochondria. A) Immunoblot analysis of SLC7A5 and mitochondrial cytosol β‐tubulin, mitochondrial outer membrane TOM20, and mitochondrial inner membrane COX4 in RAW264.7 cell subcellular fractionations. B,C) Immunofluorescent staining of MitoTracker and EGFP (SLC7A5‐EGFP) in RAW264.7 cells (B) and MitoTracker and SLC7A5 in BMDMs (C). Scale: 20 µm (co‐localization analyses are shown on the right). D) Schematic of the experimental design of label‐free proteomics sequencing of co‐IP products in SLC7A5‐FLAG‐transfected RAW264.7 cells (Anti‐FLAG versus anti‐IgG. *n* = 3/group). E) Co‐IP of SLC7A5‐FLAG‐transfected RAW264.7 cells with FLAG antibody followed by immunoblot analysis with SLC7A5 or PGAM5 antibodies. F) PLA signal (red) from SLC7A5‐FLAG‐ and PGAM5‐HA‐ or sh‐*Pgam5*‐transfected RAW264.7 cells (*n* = 13 visual fields per group, each containing over ten cells). Scale: 20 and 5 µm (high magnification). G) Co‐IP of HA (PGAM5‐HA) and immunoblot (IB) analysis of FLAG (SLC7A5‐FLAG) in RAW264.7 cells. H) Co‐IP of FLAG (SLC7A5‐FLAG) and immunoblot (IB) analysis of HA (PGAM5‐HA) and FLAG (SLC7A5‐FLAG) in the cytosol and mitochondrial fractions of RAW264.7 cells. I) Representative images of PLA signal (green) between HA (PGAM5‐HA) and FLAG (SLC7A5‐FLAG) and Mitotracker deep red staining in RAW264.7 cells. Scales: 20 µm and 2 µm (high magnification). J) Key hydrophobic residues (colored as magenta spheres) that stabilize the SLC7A5‐PGAM5 complex in model 2. K) Schematic of full‐length PGAM5 (PGAM5‐HA) and truncation (ΔPGAM5‐HA). L) Co‐IP of SLC7A5 and HA in vehicle‐, *Pgam5*‐HA‐, and Δ*Pgam5*‐HA transfected HEK293T.

The immunoblot analysis showed that SLC7A5 was expressed in mitochondria from RAW264.7 cells (**Figure** **6**A). Immunofluorescent staining visualized SLC7A5 expression in Mito‐tracker‐positive mitochondria in RAW264.7 cells (Figure 6B) and BMDMs (Figure 6C). To delineate the molecular basis of SLC7A5‐mediated alteration of mitochondrial function, we expressed the SLC7A5‐FLAG protein in RAW264.7, using the FLAG antibody to precipitate SLC7A5‐FLAG‐associated proteins, followed by mass‐spectrometry (Figure 6D). The significant enrichment of WikiPathways (Figure S8A, Supporting Information) and cellular localization (Figure S8B, Supporting Information) in the SLC7A5‐FLAG immuno‐complex identified PGAM5, localized in the mitochondrial membrane, as a potential interacting protein with SLC7A5. A direct SLC7A5‐FLAG interaction with both endogenous PGAM5 (Figure 6E) and PGAM5‐HA (Figure 6F,G) was verified by co‐immunoprecipitation (co‐IP) (Figure 6E,G) and proximity ligation assays (PLAs) (Figure 6F) in RAW264.7 cells transfected with SLC7A5‐FLAG. Co‐IP following mitochondrial fractionation (Figure 6H) and PLA co‐stained with MitoTracker (Figure 6I) confirmed the interaction between SLC7A5‐FLAG and PGAM5 in the mitochondria fraction. We further employed computational modeling to provide structural evidence that SLC7A5 and PGAM5 could bind to the mitochondrial membrane (Figure 6J; Figure S8C–E, Supporting Information). SLC7A5 may bind to PGAM5 through transmembrane helix interactions, and we built two SLC7A5‐PGAM5 complex models (Figure S8C, Supporting Information). After conducting 500 ns all‐atom molecular dynamics (MD) simulations of the complex in a POPC lipid membrane, model 2 exhibited a remarkably high number of inter‐protein contacts and low root mean square fluctuations (RMSF) (Figure S8D, Supporting Information), both indicating a stable SLC7A5‐PGAM5 complex. The lateral atom density projection plot further shows that PGAM5 in model 2 was more closely packed with SLC7A5 compared to model 1 (Figure S8E, Supporting Information). Superior stability of model 2 was attributed to the multiple hydrophobic interactions between PGAM5 and SLC7A5 (Figure 6J). Our modeling indicated that SLC7A5 and PGAM5 can form stable complexes in the membrane environment via transmembrane helix packing. To validate this, we further truncated the full‐length PGAM5 based on the predicted binding site and transfected domain (ΔPGAM5‐HA, residues 30–289) into HEK293T cells (Figure 6K). Co‐IP results indicated that the 1‐29 region of PGAM5 is critical for binding SLC7A5 (Figure 6L).

### 2.6 PGAM5 Ameliorates SLC7A5‐Induced Mitochondrial Dysfunction Through Interaction with NDUFV1

**Figure 7:**
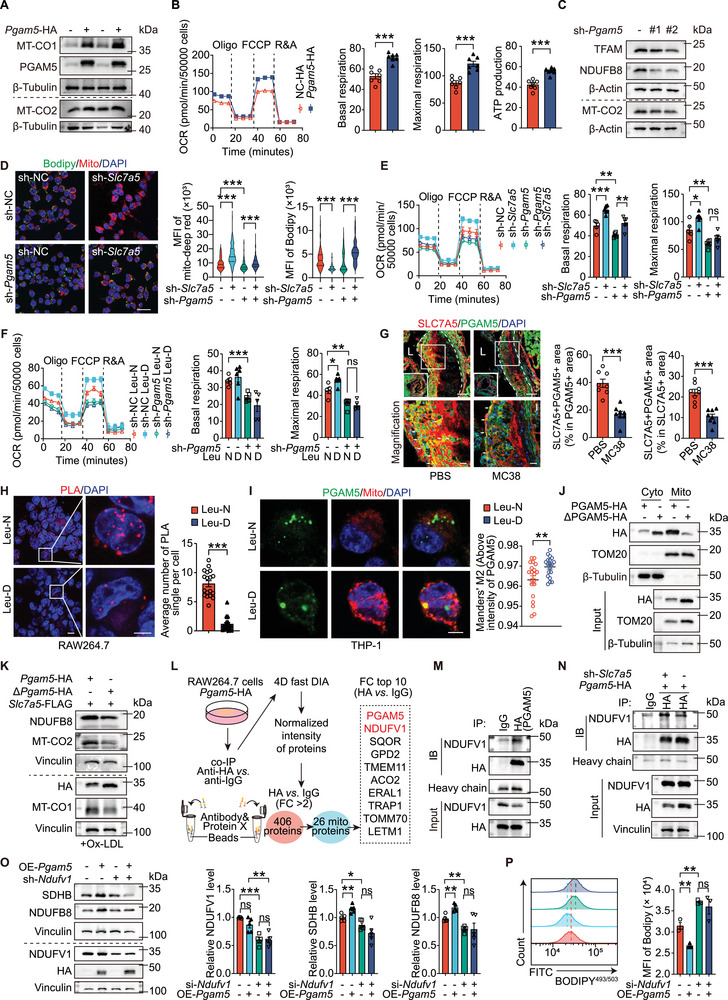
PGAM5 ameliorates SLC7A5‐induced mitochondrial dysfunction through interaction with NDUFV1. A) Immunoblot analysis of OXPHOS‐related protein MT‐CO1 and PGAM5 in *Pgam5*‐HA‐transfected RAW264.7 cells. B) OCR of RAW264.7 cells that were transfected with PGAM5‐HA or NC‐HA (*n* = 7/group). C) Immunoblot of OXPHOS‐related proteins (MT‐CO2 and NDUFB8) in RAW264.7 cells transfected with three *Pgam5*‐knockdown sequences (*n*≥5/group). D) Immunofluorescence staining of BODIPY^493/503^ and MitoTracker‐deep red in RAW264.7 cells with the indicated treatment (*n*≥100 cells/group). Scale: 20 µm. E) OCR in RAW264.7 cells transfected with or without sh‐*Slc7a5* or sh‐*Pgam5* (*n* = 5‐6/group). F) OCR in RAW264.7 cells transfected with sh‐NC or sh‐*Pgam5* in Leu‐N and Leu‐D media and treated using ox‐LDL for 24 h (*n* = 5‐6/group). G) Immunofluorescent staining of SLC7A5 (red) and PGAM5 (green) in the aortic sinus of *Apoe* ^−/−^ mice that received PBS or MC38. SLC7A5^+^PGAM5^+^ areas were quantified (*n* = 7‐8 mice/group). Scale: 100 µm and 20 µm (high magnification). H) PLA detection of interaction between endogenous SLC7A5 and PGAM5 in macrophages derived from THP‐1 cells, with or without leucine deficiency (*n* = 18‐19 visual fields per group, each containing over 30 cells). Scale:10 µm and 5 µm (high magnification). I) Immunofluorescent staining of PGAM5 and MitoTracker in THP‐1‐derived macrophages after 24‐h ox‐LDL treatment with or without 12‐h leucine deficiency (*n*≥100 cells from 19‐20 visual fields per group). Scale: 5 µm. J) Immunoblotting of subcellular fractions from HEK293T cells transfected with *Pgam5*‐HA‐ or Δ*Pgam5*‐HA, probed for HA, β‐tubulin (cytosolic marker), and TOM20 (mitochondrial outer membrane marker). K) *Pgam5*‐HA‐ or Δ*Pgam5*‐HA‐transfected SLC7A5‐overexpression RAW264.7 cells treated with ox‐LDL for 24 h. Immunoblotting of NDUFB8, MTCO1, and MTCO2 (*n* = 3‐4/group). L) Schematic of the experimental design of proteomics sequencing of co‐IP products in *Pgam5*‐HA‐transfected RAW264.7 cells (Anti‐HA versus anti‐IgG). M) Co‐IP of PGAM5‐HA‐transfected RAW264.7 cells with HA antibody followed by immunoblot analysis with HA or NDUFV1 antibodies. N) Co‐IP of PGAM5‐HA‐transfected RAW264.7 cells with or without SLC7A5 knockdown. O) Immunoblot of OXPHOS‐related proteins (SDHB and NDUFB8) in RAW264.7 cells transfected with or without OE‐*Pgam5* or si‐*Ndufv1* (*n* = 5/group). P) Flow cytometry of BODIPY^493/503^ in indicated RAW264.7 cells treated with ox‐LDL(*n* = 4/group). Data are mean ± SEM. ns, not significant, *P<0.05, **P<0.01, ***P<0.001, unpaired Student's *t*‐test (B, E‐I, O, and P), Kruskal‐Wallis test (D).

To investigate the effect of PGAM5 on mitochondrial function, we generated PGAM5‐overexpressing (*Pgam5*‐HA) RAW264.7 cells. Overexpression of exogenous PGAM5 increased mitochondrial function and mass in macrophages (**Figure** **7**A,B, Figure S9A,B, Supporting Information), but also increased glycolytic capacities (Figure S9C, Supporting Information). Knockdown of PGAM5 with shRNA significantly reduced TFAM and OXPHOS protein levels in RAW264.7 cells (Figure 7C; Figure S9D, Supporting Information), suggesting PGAM5 alleviates mitochondrial dysfunction. To assess whether PGAM5 can reduce mitochondrial dysfunction caused by SLC7A5, sh‐*Slc7a5* RAW264.7 cells were transduced with the lentivirus encoding sh‐*Pgam5*. *Slc7a5* knockdown elevated mitochondrial mass and decreased the BODIPY^493/503^‐positive areas in RAW264.7 cells, and *Pgam5* knockdown reversed these beneficial functions of *Slc7a5*‐knockdown (Figure 7D). Similarly, *Slc7a5*‐knockdown elevated mitochondria functions, including basal respiration, maximal respiration, and ATP production. *Pgam5* knockdown partially blocked the beneficial effects of *Slc7a5*‐knockdown (Figure 7E). Therefore, *Slc7a5* knockdown reduced foam cell formation via PGAM5‐mediated mitochondrial function. Leucine deficiency increased the maximal respiration rate but not basal respiration or ATP production. *Pgam5*‐knockdown (sh*‐Pgam5*) reduced all the variables. However, in RAW264.7 cells with *Pgam5*‐knockdown, leucine deficiency did not affect maximal respiration rate (Figure 7F). These observations suggest that leucine deficiency promotes mitochondrial function and decreases lipid accumulation, partially via PGAM5.

To elucidate how PGAM5 reduces foam cell formation and mitochondrial dysfunction under leucine sufficient, we observed that neither SLC7A5 overexpression nor deficiency altered PGAM5 expression (Figure S9E,F, Supporting Information), suggesting mechanisms beyond regulation of protein expression. Immunofluorescent staining revealed significantly diminished co‐localizations of SLC7A5 and PGAM5 in plaques from tumor‐bearing mice (Figure 7G). Hence, we hypothesized that the leucine mediates the SLC7A5‐PGAM5 interaction within the plaque. Consistent with this, PLA signals showed that the interaction of SLC7A5 and PGAM5 reduced in SLC7A5‐FLAG and PGAM5‐HA co‐transfected RAW264.7 cells under leucine deficiency (Figure 7H). We validated this by co‐IP in H293T cells, a human epithelial cell line (Figure S9G, Supporting Information), and co‐localization in macrophages derived from THP‐1 cells, a human macrophage cell line (Figure S9H, Supporting Information). Immunofluorescent with MitoTracker showed increased mitochondrial localization of endogenous PGAM5 in leucine‐deprived THP‐1 cells (Figure 7I), and similarly in *Slc7a5*‐deficient versus sufficient BMDMs under ox‐LDL (Figure S9I, Supporting Information). These findings implicate enhanced mitochondrial location of PGAM5 in improved mitochondrial function and reduced lipid accumulation. Mitochondrial fractionation confirmed that full‐length PGAM5‐HA (containing the 1–29 region) resides primarily in mitochondria, whereas ΔPGAM5‐HA is cytosolic (Figure 7J), consistent with the 1–29 region mediating both SLC7A5 binding (Figure 6J–L) and mitochondrial anchoring.^[^ ^36^ ^]^ Functionally, in SLC7A5‐overexpressing RAW264.7 cells, ΔPGAM5‐HA yielded reduced mitochondrial protein under ox‐LDL than full‐length PGAM5‐HA, highlighting the importance of PGAM5 mitochondrial localization for normal OXPHOS function in macrophages (Figure 7K; Figure S9J, Supporting Information).

To elucidate how PGAM5 maintains mitochondrial homeostasis, we performed proteomic analysis of PGAM5 immunoprecipitates, identifying 26 mitochondrial proteins (fold change>2, HA versus IgG), among which NDUFV1 exhibited the highest fold change (Figure 7L). Co‐IP suggested that NDUFV1, a core subunit of NADH dehydrogenase (Complex I) in the mitochondrial respiratory chain, binds PGAM5 (Figure 7M), and this interaction was further enhanced in SLC7A5‐knockdown macrophages (Figure 7N), suggesting that SLC7A5 regulates the PGAM5–NDUFV1 interaction. To confirm the role of NDUFV1 in PGAM5 alleviating mitochondrial dysfunction, OE‐*Pgam5* RAW264.7 cells were transduced with si‐*Ndufv1* (small interfering RNA). Compared with the control siRNA, NDUFV1 knockdown significantly reduced OXPHOS protein (SDHB and NDUFB8) levels (Figure 7O) and mitochondrial membrane potential (Figure S9K, Supporting Information), and increased lipid accumulation under ox‐LDL (Figure 7P). These changes abolished the PGAM5‐induced increases in OXPHOS protein levels (Figure 7O) and reductions in lipid accumulation (Figure 7P). Notably, PGAM5 overexpression did not alter NDUFV1 protein levels (Figure 7O).

### 2.7 Slc7a5 Deficiency Increases CD5L^hi^ Macrophages by Restoring Mitochondrial Function

**Figure 8:**
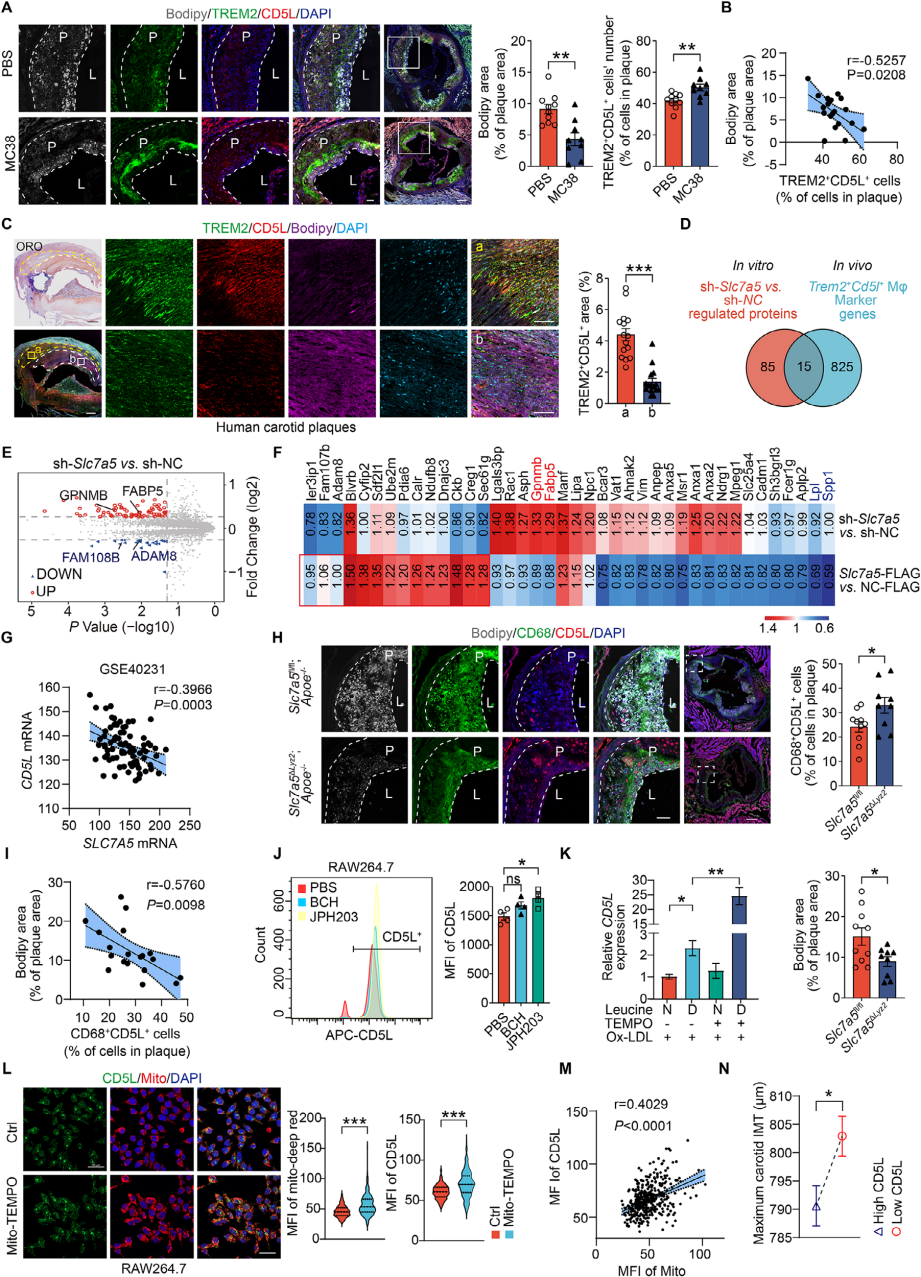
*Slc7a5* deficiency increases CD5L^hi^ macrophages by restoring mitochondrial function. A) Immunofluorescence staining and quantification of TREM2^+^CD5L^+^ cells and BODIPY^493/503^ in aortic sinus plaques from PBS‐ and MC38‐treated *Apoe* ^−/−^ mice (*n =*9‐10/group). Scale: 100 µm, inset: 20 µm. B) Correlation between the proportion of TREM2^+^CD5L^+^ cells and BODIPY^493/503^ area in plaques from indicated mice (*n =*19). C) TREM2^+^CD5L^+^ cells in human carotid plaques and their lipid content measured using ORO and BODIPY^493/503^ high (a, white dotted) and low (b, yellow dotted) areas. Each point represents a 0.2 mm^2^‐area. Pooled quantifications from 3 human plaques. Scale: 500 and 100 µm (high magnification) D) Venn diagram illustrating the common genes of differentially‐expressed proteins (sh‐*Slc7a5* versus sh‐NC, FC≥1.2 or FC≤0.83, *P*<0.05) in RAW264.7 cells and marker genes of the *Trem2* ^+^ *Cd5l* ^+^ macrophages. E) Volcano plot illustrating *Trem2* ^+^ *Cd5l* ^+^ macrophage marker genes in the proteome from RAW264.7 cells after sh‐*Slc7a5* versus sh‐NC (FC≥1.2 or FC≤0.83, *P*<0.05). F) Heatmap indicating the fold change in expression of *Trem2* ^+^ *Cd5l* ^+^ macrophage marker genes in the proteomics of RAW264.7 cells with *Slc7a5* knockdown or overexpression. Numbers represent fold change compared to the respective control group (*n =*3). G) Correlation analyses of *SLC7A5* and *CD5L* mRNA levels in human carotid atherosclerotic plaques (GSE43292; *n* = 64). H) Immunofluorescent staining of BODIPY^493/503^, CD68, and CD5L in aortic sinus from 16‐week HFD‐fed *Slc7a5* ^fl/fl^ *; Apoe* ^−/−^ mice and *Slc7a5* ^fl/fl^ *Lyz2*‐cre; *Apoe* ^−/−^ mice, and quantification of CD68^+^CD5L^+^ cells and BODIPY^493/503^ area (*n* = 9‐10 mice per group). Scale: 200 µm and 50 µm (high magnification). I) Correlation analysis between the proportion of CD68^+^CD5L^+^ cells and BODIPY^493/503^ area in indicated two groups of mice (*Slc7a5* ^fl/fl^ *; Apoe* ^−/−^ and *Slc7a5* ^fl/fl^ *Lyz2*‐cre; *Apoe* ^−/−^) (*n* = 19 mice). J) Flow cytometry detection and quantification of CD5L expression in BCH or JPH203‐treated RAW264.7 cells (*n =*4). K) Quantitative RT‐PCR analysis of *Cd5l* expression in RAW264.7 cells treated with ox‐LDL with or without mito‐TEMPO for 24 h in Leu‐N or Leu‐D media (*n =*3/group). L) Immunofluorescent staining of CD5L and MitoTracker‐deep red (*n*≥100 cells/group). Scale: 20 µm. M) Pearson's correlation of mean fluorescence intensity (MFI) of CD5L and MitoTracker in RAW264.7 cells treated with or without mito‐TEMPO for 24 h (*n*>200 cells). N) Least‐squares means of maximum carotid IMT in UK Biobank participants stratified by high versus low plasma CD5L levels (*n* = 2284/group). Data are mean ± SEM. ns, not significant, *P<0.05, **P<0.01, ***P<0.001, unpaired Student's *t*‐test (A, C, H and J), Pearson's correlation test (B, G, I and M), one‐way ANOVA with Dunnett correction (K), Mann‐Whitney test (L), and *t*‐test between least squares means (N).

To confirm that CD5L^hi^ macrophages in plaques represent a metabolically reprogrammed subset with stronger mitochondrial function and reduced lipid accumulation, we analyzed the cellular distribution of the identified markers using immunofluorescence staining in mouse and human atherosclerotic plaques. The number of CD5L^+^TREM2^+^ cells was higher, and BODIPY^493/503^‐stained lipid droplets areas were lower in aortic sinus atherosclerotic plaques from MC38 tumor‐bearing mice (**Figure** **8**A). The number of these macrophages negatively correlated with the BODIPY^493/503^ area (Figure 8B). Similarly, TREM2^+^CD5L^+^ regions exhibited a lower lipid content than other regions in human carotid atherosclerotic plaques (Figure 8C). Therefore, an increase in the percentage of *Cd5l* ^+^ macrophages negatively correlates with lesion lipid deposition.

Next, we explored the *Slc7a5*‐knockdown effect on macrophage subset. Fifteen MP5‐marker genes encoding proteins were identified among those differentially expressed in *Slc7a5*‐knockdown RAW264.7 cells (Figure 8D), including *Gpnmb* and *Fabp5* (Figure 8E), with strong specificity in MP5 (Figure S3B, Supporting Information). Furthermore, lipoprotein lipase (LPL) and secreted phosphoprotein 1 (SPP1) expressions decreased in OE‐*Slc7a5* cells (Figure 8F), suggesting that *Slc7a5*‐knockdown reprogrammed macrophages toward MP5 phenotype. A weak but significant negative correlation was observed between *SLC7A5* and *CD5L* mRNA expression in human atherosclerotic plaques (Figure 8G). Immunofluorescent staining revealed significantly elevated levels of aortic sinus plaque CD68^+^CD5L^+^ macrophage and reduced plaque foam cell (BODIPY^493/503^) contents in *Slc7a5* ^fl/fl^ *Lyz2*‐cre; *Apoe* ^−/−^ mice compared with their littermate (Figure 8H). Plaque CD68^+^CD5L^+^ macrophage contents negatively correlated with plaque BODIPY^493/503^ areas (Figure 8I). Therefore, SLC7A5 deficiency in macrophages increases CD5L^+^ macrophages content in atherosclerotic plaque. In vitro, pharmacological inhibition of SLC7A5 with JPH203 (but not BCH) (Figure 8J), leucine deprivation (Figure 8K), or mitochondrial activation with mito‐TEMPO (Figures 7L) up‐regulated CD5L expression in ox‐LDL‐treated macrophages. This increase positively correlated with mitochondrial content (Figure 8M), indicating that SLC7A5‐dependent leucine transport regulates CD5L expression through mitochondrial metabolic reprogramming.

Given that CD5L is a secreted glycoprotein, we also assessed the relationship between its plasma levels and AS. Since carotid intima‐media thickness (IMT) is a strong predictor of future cerebral and cardiovascular events and is a widely used surrogate marker for AS,^[^ ^37^ ^]^ we evaluated the association between plasma CD5L protein levels and carotid IMT using the UKB database. After stratifying 4568 participants into High‐CD5L (n = 2284) and Low‐CD5L (*n* = 2284) groups, analyses adjusted for age and sex showed that the least squares mean maximum carotid intima‐media thickness (IMT) was significantly lower in the high‐CD5L group than in the low‐CD5L group (790.57±3.546 µm versus 802.91±3.528 µm, *P* = 0.014) (Figure 8N), suggests that high plasma CD5L level may be a protective factor against AS.

## 3 Discussion

AS is the leading cause of myocardial infarction and stroke, accounting for >25% of all‐cause mortality.^[^ ^38^ ^]^ Despite significant progress in preventive strategies and pharmacotherapy, residual risks still exist, highlighting the need for a better understanding of AS progression and the development of molecular therapies. Recent research has focused on the role of elevated plasma BCAAs resulting from high‐protein diets^[^ ^4^, ^9^ ^]^ and the impact of defects in BCAA metabolism^[^ ^13^ ^]^ in AS progression. Herein, we confirmed the role of excessive leucine in aggravating AS progression by increasing the number of apoptotic macrophages in the plaques.

Intriguingly, we observed markedly reduced plasma leucine levels in both tumor‐bearing patients and mice, and subcutaneous tumor models in atherosclerotic mice exhibited decreased atherosclerotic lesion formation. Although tumors are typically pro‐inflammatory, these results highlight how systemic leucine depletion can reshape the metabolic microenvironment relevant to AS. Plasma leucine largely reflects dietary intake and proteolytic flux,^[^ ^9^, ^39^, ^40^ ^]^ and altered BCAA profiles‐including reduced leucine in cachexia or severe malnutrition‐are observed in human disease (e.g., lean versus obese differences).^[^ ^41^ ^]^ Although tumor‐induced cachexia does not represent the typical etiology of AS, it models a clinically relevant low‐leucine metabolic state (cachexia, advanced chronic disease, severe malnutrition) and thus provides biological insight complementary to studies linking elevated BCAAs with cardiometabolic risk. Importantly, leucine repletion in our model abolished the tumor‐associated atheroprotective effect, supporting a causal role for leucine availability. We acknowledge that tumor‐associated inflammation and hormonal changes may confound this model; future work using dietary BCAA manipulation, tissue‐specific metabolic models, and human tissue or cohort analyses will be important to confirm translational relevance.

Using various methods to induce leucine deprivation, the mitochondrial function of macrophages in the atherosclerotic microenvironment is metabolically reprogrammed, thereby reducing lipid accumulation and mitigating AS. First, we minimized the confounding effects of inflammation, blood lipids, and other factors on plaque progression in a mouse model of advanced AS with concomitant MC38 tumors. Nevertheless, despite the inevitably complex tumor microenvironment, our study confirmed that tumor‐driven leucine deprivation reduces atherosclerotic lesions. Regrettably, these experimental findings are challenging to validate clinically. Although clinical data indeed indicate elevated cardiovascular disease (CVD) mortality risk in patients with cancer,^[^ ^42^ ^]^ this association is often confounded by treatment‐related factors, such as radiotherapy, chemotherapy, and surgery.^[^ ^43^ ^]^ Moreover, compared with individuals without cancer, the increased CVD risk in patients with confirmed malignancy is primarily driven by heart failure and venous thromboembolism, whereas the risks of ischemic heart disease and stroke do not appear to be elevated.^[^ ^44^, ^45^ ^]^ This is not contradictory to our animal results. Second, we used the selective leucine transporter SLC7A5 inhibitor JPH203 and a Leu‐KO high‐fat diet to treat advanced AS in *Apoe* ^–/−^ mice. JPH203, developed as a potential clinical antitumor drug,^[^ ^14^, ^15^ ^]^ inhibits leucine transport into cells, with clinical efficacy in patients with biliary tract cancer and a good safety profile and high tolerance.^[^ ^46^ ^]^ This finding has significant clinical value for the expansion of drug indications. Finally, we investigated the effect of macrophage‐specific leucine deficiency in a mouse model. Notably, macrophage‐specific SLC7A5 deficiency in *Apoe* ^−/−^ mice alleviated AS. We identified the role of SLC7A5 in impairing mitochondrial function in foamy macrophages via SLC7A5‐PGAM5 interaction. Knockdown of SLC7A5 increased the direct interaction between PGAM5 and NDUFV1. Given that complex I (NADH dehydrogenase) is catalytic only in its active conformation,^[^ ^47^ ^]^ the enhanced PGAM5‐NDUFV1 binding may influence mitochondrial function. This effect could be mediated by NDUFV1's association with other complex I subunits, highlighting a novel therapeutic target for advanced AS.

Our data supports the elevated plasma leucine aggravates atherosclerotic plaque instability in advanced AS. Though one study reported that supplementing leucine or BCAAs in *Apoe* ^−/−^ mice reduces AS,^[^ ^48^ ^]^ those mice were on chow and treated early in disease, when plaque macrophage metabolism differs from advanced AS (see spatial transcriptomics). Conversely, impaired BCAA catabolism causes aortic BCAA accumulation, mitochondrial ROS, and inflammation.^[^ ^13^ ^]^ With BCKDHA deficiency, leucine accumulation further disrupts mitochondrial and metabolic functions in macrophages. Optimal leucine deficiency can reprogram macrophage metabolism and enhance mitochondrial function.

To investigate whether monocyte‐macrophages reprogrammed by subcutaneous MC38 tumors contribute to AS progression, we used single‐cell and spatial transcriptomics to map their subsets, expression profiles, and spatial distribution in mouse aortas. Our analysis revealed increased expression of the proliferation gene *Mki67* in aortic monocytes, consistent with the rise in activated circulating monocytes in mice on a leucine‐deficient diet. Despite this monocyte activation, AS was alleviated, indicating that the macrophage metabolic shift was the dominant factor. The macrophages reprogrammed under leucine deficiency exhibited enhanced mitochondrial function, marked by high CD5L expression, reduced foam‐cell lipid content, and stabilized atherosclerotic plaques in vitro and in vivo, resulting from leucine deficiency. CD5L^hi^ macrophage or elevated circulating CD5L may be protective factors against AS. ScRNA‐seq further identified a *Trem2* ^+^ *Cd5l* ^+^ macrophage subcluster, exhibiting enhanced cellular function in the presence of tumors, with a metabolic status mirroring the overall plaque metabolic alteration. Reportedly, *TREM2* ^hi^ macrophages exhibit high expression of genes involved in cholesterol metabolism, fatty acid transport, OXPHOS, proteasome and lysosomal activity and PPAR signaling.^[^ ^49^ ^]^ TREM2 is crucial for lipid uptake by macrophages, promotes foam cell survival, effectively removes dead cells from tissue, and may significantly contribute to increasing plaque stability.^[^ ^50^ ^]^ CD5L induces lipolysis and reduce lipid droplet size in adipocytes.^[^ ^31^, ^51^ ^]^ However, excessive CD5L can recruit macrophages to adipose tissue and trigger chronic inflammation.^[^ ^51^ ^]^ Moreover, a substantial fraction of circulating CD5L is misfolded and functionally limited,^[^ ^52^ ^]^ warranting caution regarding dose and indications for therapeutic use. Consistent with this framework, CD5L^hi^ macrophages were increased in MC38 plaques and inversely correlated with lipid deposition in advanced lesions, supporting a protective metabolic shift induced by leucine deprivation.

Our multi‐transcriptome analyses revealed metabolic changes during the transition from normal arterial wall to advanced AS. Under physiological conditions, contractile SMCs and ECs mainly use OXPHOS and glycolysis, respectively.^[^ ^53^ ^]^ In early AS or relatively lipid‐poor stable plaques, monocytes enter the intima, become macrophages that rely on OXPHOS, and SMCs switch to a synthetic state with elevated OXPHOS to support biosynthesis and energy needs. to phagocytose lipids and transform into macrophages.^[^ ^54^ ^]^ Therefore, the global OXPHOS level increases in early atherosclerotic plaques compared with that in normal arteries. Following AS progression, exacerbated hypoxia within plaques, increased lipid accumulation, and chronic inflammation cause cellular reprogramming and a shift from oxidative metabolism to anaerobic glycolysis.^[^ ^55^ ^]^ Therefore, in addition to increased glycolytic activity, aerobic metabolic pathways, such as fatty acid degradation and the TCA cycle, are diminished in the plaques of advanced AS. Our spatial transcriptomics indicates that this reprogramming pattern is altered in plaques when tumors are comorbid.

Our study has some limitations. SLC7A5, which transports leucine across the cell membrane, localizes to the mitochondrial membrane and regulates mitochondrial mass and function by interacting with PGAM5. However, whether SLC7A5 translocates leucine into the mitochondria or influences BCAA metabolism remains unclear and should be explored in future research.

In conclusion, we uncovered a mechanism by which SLC7A5 deficiency mediates the improvement of mitochondrial function in macrophages, illustrated how leucine deficiency induces metabolic reprogramming of macrophages during AS progression through multi‐omics analysis and experimental verification. These findings suggest that therapies by mitochondrial activation and targeting SLC7A5 may represent novel strategies for treating advanced AS.

## 4 Experimental Section

### Cohorts in UKB Database

For the follow‐up study of AMI, baseline data was collected from ≈500000 individuals in the UK between 2006 and 2010, with a follow‐up period of 12‐16 years (13.70 ± 2.136 years). During the follow‐up, a total of 252128 participants were included in the study, excluding patients with clinically diagnosed coronary heart disease, stroke, prior heart failure, atrial fibrillation, or cardiomyopathy. The baseline characteristics of the cohort are presented in Table S1 (Supporting Information). Participants were divided into five groups based on plasma leucine levels, ranked from low to high as Leu‐0, Leu‐1, Leu‐2, Leu‐3, and Leu‐4. After adjusting for age and sex, COX regression analysis was performed to examine the relationship between plasma leucine levels and the incidence of new‐onset AMI during follow‐up. For the cross‐sectional study of plasma BCAA levels, a total of 102056 participants were included with available plasma BCAA levels and baseline data, and a 1:1 PSM by age and sex was performed on 8226 participants with cancer. paired Student's *t*‐test analyses were performed between the two groups. The baseline characteristics of the cohort are presented in Table S2 (Supporting Information). For the analysis of carotid IMTs in groups with different plasma CD5L level, we included a total of 4351 participants with available plasma CD5L protein levels and carotid intima‐media thickness (IMT) data. Plasma CD5L protein expression was obtained from the UK biobank Pharma Proteomics Project (UKB‐PPP) and quantified using Normalized Protein eXpression (NPX).^[^ ^56^ ^]^ Least squares means were obtained from a linear regression model adjusting for age and sex, and the difference between the two groups was tested using a *t*‐test.

### Human Sample Collection

The carotid artery and femoral artery of thigh samples were collected from patients who underwent a carotid endarterectomy or amputation procedure at the second Affiliated Hospital of Harbin Medical University (Heilongjiang, China), which were approved by institutional review committees and obtained following informed consent.

### Mice

Male *Apoe* ^−/−^, *Ldlr* ^−/−^, and C57Bl/6J mice (6–8 weeks) were obtained from the Cyagen Biosciences. Myeloid‐specific *Slc7a5* knockout mice were generated on a C57BL/6J background using CRISPR/Cas9, targeting exon 3 of *Slc7a5* (NCBI Reference Sequence: NM_01 1404; Ensembl: ENSMUSG00000040010) for floxing, with deletion driven by Lyz2‑Cre. Breeding strategy: *Slc7a5* ^flox/flox^ × *Lyz2* Cre^+/−^ mice (obtained from the Cyagen Biosciences) →*Lyz2* Cre^+/−^; *Slc7a5* ^flox/flox^; then crossed with *Apoe* ^−/−^ to obtain *Slc7a5* ^flox/flox^ *Apoe* ^−/−^mice. Genotyping by PCR and sequencing confirmed the knockout. Knockout mice showed no behavioral or breeding abnormalities compared with wild‑type C57BL/6. Mice for atherosclerosis study were fed with a high‐fat diet (20% protein, 40% fat, 1.25% cholesterol, D120108C, Keao Xieli Feed Co., Ltd,) for 16 weeks. For leucine restriction diet, *Apoe* ^−/−^ mice were fed for 4 weeks with either a normal leucine diet (1.6% leucine; SYSEBIO) or a leucine‑restricted diet (0% leucine; SYSEBIO) after 12 weeks on a high‐fat diet (D12079B). For mitochondrial activators treatment, *Apoe* ^−/−^ mice were randomly assigned to receive intraperitoneal injections of MitoTEMPO (MCE, Cat# HY‐112879, 0.8 mgkg^−1^ body weight) or saline twice weekly for 8 weeks during the HFD feeding period. After indicated weeks, mice were anesthetized using ketamine/xylazine, and tissues were harvested. All animal experiments have been approved by the Research Ethics Committee of the Second Affiliated Hospital of Harbin Medical University. All mice were maintained on a 12‐h light/dark schedule in a specific pathogen‐free animal facility in individually ventilated cages and were given food and water ad libitum. Ambient temperature in the animal facility was 20–24 °C, and relative humidity was 45–65%.

### Cell Culture

The MC38 mouse colon adenocarcinoma cell lines were cultured in RPMI 1640 medium (Gibico) with 10% fetal bovine serum (FBS) (Sigma, F0193) at 37 °C in a 5% CO2 atmosphere. The mouse mononuclear macrophage cell line (RAW 264.7), purchased from Cell Bank, Chinese Academy of Sciences, was cultured in DMEM described above in a 5% CO2 atmosphere at 37 °C. The human mononuclear macrophage cell line (THP‐1 cells) was cultured in RPMI 1640 supplemented with 10% FBS in a 5% CO2 atmosphere at 37 °C. Cells were tested negative for mycoplasma infection using the Mycoplasma Detection Kit (Beyotime, #C0297S).

To generate bone marrow‐derived macrophages (BMDMs), bone marrow was isolated from 8 to 12‐week‐old mice and cultured for 7‐10 days in DMEM, supplemented with 10% FBS, 100 UmL^−1^ penicillin, 0.1 mgmL^−1^ streptomycin, and 20% L‐929 mouse fibroblast‐ conditioned medium (L‐cell‐conditioned medium). L‐cell‐conditioned medium was obtained by growing L‐929 cells (American Type Culture Collection) in the DMEM described above. Cells were cultured in a humidified CO2 incubator at 37 °C.

To establish stably transfected cell lines, RAW264.7 cells were transfected with lentivirus containing the following: sh‐NC, sh‐*Slc7a5*‐1, sh*‐Slc7a5*‐2, sh*‐Slc7a5*‐3, sh‐NC, sh‐*Pgam5*‐1, sh*‐Pgam5*‐2, sh*‐Pgam5*‐3, OE‐NC‐FLAG, OE‐*Slc7a5*‐FLAG, OE‐NC‐GFP, OE‐ *Slc7a5*‐GFP, OE‐NC‐HA, OE‐*Pgam5*‐HA. 48 h after transfection, cells were selected with puromycin (4 µgml^−1^) or G418 (1 mgmL^−1^).

### Atherosclerotic Mouse Model with Tumor

6 to 8‐week‐old male *Apoe* ^−/−^ mice, and *Ldlr* ^−/−^ mice were fed with HFD for 12 weeks (as mentioned above). After 12 weeks, test group mice received subcutaneous injection of 100 µL PBS containing 5 × 10^4^ MC38 cells. The normal group received a subcutaneous injection of the same volume of PBS. Daily food intake was measured for each group. Mice were sacrificed after 4 weeks, and maintained an HFD during this period.

### Leucine Excessive Supplementation in Mice

The 8‐week‐old *Apoe* ^−/−^ male mice were fed a high‐fat diet with free access to water throughout the procedure, as described previously, for 16 weeks. During the last 4 weeks, the water containing excess leucine (Sigma, Cat# L8912, 8 mgml^−1^, approach to 1.6 mgg^−1^ body weight) was treated in the animals as described in our recent study. This dose was based on previous reports without affecting food intake or weight gain in mice.^[^ ^13^, ^57^ ^]^ The mice in the control group were fed with water. Additionally, the physical conditions of the mice were observed every day. All mice that died during the experiment were autopsied immediately.

### Analysis of the Degree of Atherosclerotic Lesions in Mice

Atherosclerotic lesion analysis adhered to the guidelines for experimental AS studies described in the American Heart Association statement.^[^ ^58^ ^]^ The mice were euthanized and irrigated with saline from the left ventricle. The full‐length aortas and hearts were carefully separated from the fat and dissected out, then fixed with 4% PFA for 24 h. Washed and stained aortas with Oil red O working solution, the proportion of Oil red O positive area to the total vascular area was calculated.

The procedure for making a section of the aortic root (including the frozen section and paraffin section) was followed by previous studies.^[^ ^59^ ^]^ Histological analysis was performed on serial paraffin cross sections (4.5 µm) or frozen sections (6 µm). For aortic root section, three serial sections were selected at 80‐µm intervals starting from the first appearance of the three valve leaflets. For brachiocephalic trunk section, three serial sections were selected at 100‐µm intervals starting from the junction of the aortic arch and the brachiocephalic trunk. Aortic lesion size was obtained by averaging the lesion areas in three slides per mouse. The plaque size, necrotic core size (identified as an acellular area, with a 3000 µm^2^ threshold applied to exclude tiny H&E‐negative areas), and fiber cap thickness (quantified by choosing the largest necrotic core from triplicate sections, measuring from the thinnest part of the cap) were evaluated by H&E staining (Solarbio, G1120) of aortic root sections. The lipid content in plaques was evaluated by Oil red O staining (Solarbio, G1261). Masson's trichrome staining (Solarbio, G1346) was used to evaluate the collagen fiber content in plaques.

### Serum Lipid Concentration Analysis

The mice were anesthetized, blood was extracted through enucleation of the eyeballs, and then cervical dislocation was performed. The serum was collected by centrifugation at 3000 x rpm for 15 min at 4 °C. Then, kits from Nanjing Jiancheng Bioengineering Institute were used to evaluate the levels of serum lipids. Triglyceride (TG), total cholesterol (TC), low‐density lipoprotein cholesterol (LDL‐C), and high‐density lipoprotein cholesterol (HDL‐C) were measured by a microplate reader according to the manufacturer's instructions.

### D‐Labeled Leucine Uptake Assays In Vivo

After 4 weeks of subcutaneous injection of PBS or MC38 cells, mice were given 1 mgg^−1^ body weight of D10‐L‐leucine (CIL, DLM‐567) by oral gavage. Blood was collected from the tail vein after 0.5, 1, and 1.5 h, respectively. Plasma (50 µL/mouse) D10‐L‐leucine content was detected by LC‐Q‐TRAP‐MS. The mice were euthanized after the last blood collection, and the aortas (50 mg/mouse) and tumors (100 mg/mouse) were removed for detection of D10‐L‐Leucine content by LC‐Q‐TRAP‐MS. The differences of D10‐L‐leucine in plasma and aortas between the PBS group and MC38 group were analyzed.

### Plasma Metabolite Extraction for LC‐MS

All 23 amino acid standards and 2 stable isotope‐labeled standards, as well as ammonium acetate, were obtained from Sigma‐Aldrich. Methanol, acetonitrile, and formic acid (Optima LC‑MS grade) were from Thermo Fisher Scientific. Ultrapure water was from Millipore. Plasma/serum samples (100 µL) were diluted tenfold with water. An aliquot (50 µL) was extracted with 200 µL acetonitrile: methanol (1:1, v/v) containing mixed internal standards by vortexing, incubated on ice for 30 min, and centrifuged at 12,000 rpm for 10 min. The supernatant was used for ultra‐high performance liquid chromatography coupled to tandem mass spectrometry (UHPLC‑MS/MS) analysis. Amino acids were quantified on an ExionLC AD coupled to a QTRAP 6500+ (AB SCIEX). Chromatographic separation was used an ACQUITY UPLC BEH Amide column (2.1×100 mm, 1.7 µm) at 50 °C, with a 0.30 mLmin^−1^ flow. Mobile phases: solvent A, 0.1% formic acid in 5 mM ammonium acetate; solvent B, 0.1% formic acid in acetonitrile. The solvent gradient was set as follows: initial 80% B, 0.5 min; 80‐70% B, 2 min; 70‐45% B, 4 min; 45‐80% B, 6.01 min; 80% B, 9 min. MS operated in positive MRM with the following settings: IonSpray Voltage (5500 V), Curtain Gas (35 psi), Ion Source Temp (550 °C), Ion Source Gas of 1 and 2 (50 and 60 psi). Analyses were performed at Novogene (Beijing, China).

### Aortic Singlet Preparation

Single‐cell suspensions of the aorta were prepared as previously reported^[^ ^60^ ^]^ with minor modifications. In brief, mice were perfused with at least 10 mL of fresh cold PBS to eliminate blood contamination before isolating the aorta. Carefully dissected and removed the perivascular fat and cardiac muscle, cut the aorta into ≈2 mm pieces, and placed aorta in an enzyme cocktail (containing collagenase I (450 units/ml), collagenase XI (250 units/ml), DNase I (120 units/ml), and hyaluronidase (120 units/ml)). After incubation at 37 °C for 45 min, the cell suspension was filtered through a 70 µm filter and centrifuged at 500 x g for 5 min. Resuspend the cells in 1 ml of tissue storage solution and assess the cell number and cell viability by trypan blue and hemocytometer.

### Single‐Cell RNA‐Sequencing (scRNA‐seq) and Quantification

Aortas from *Apoe* ^−/−^ mice (*n* = 25 per group) fed a HFD for 16 weeks were pooled and digested with an enzyme mixture for single‐cell RNA‐sequencing (scRNA‐seq) analysis of aortic CD45^+^ cells. Aortic single cells were sorted by CD45 positive magnetic beads (Miltenyi Biotec, 130‐11‐618). After nucleic acid extraction, reverse transcription, amplification, library construction, and sequencing with Illumina Novaseq 6000 platform, Cell Ranger (v7.2.0) was used to align and feature count with the raw sequences. Finally, the filtered gene expression matrix was used for the downstream analysis.

For each scRNA‐seq library, DoubletFinder^[^ ^61^ ^]^ (v2.0.3) was employed to identify and remove doublets. The number of statistically significant principal components was set as the top 20, and the threshold for doublet identification was set at 5%. To ensure data accuracy, cells were removed with a mitochondrial proportion exceeding 20% and a minimum gene count below 200.

### Integration and Clustering of scRNA Data

Two quality‐controlled single‐cell datasets were merged and the standard Seurat^[^ ^62^ ^]^ (v4.3.0) workflow was applied for normalization. The top 2000 highly variable genes was selected for standardization and performed PCA dimension reduction. Subsequently, the Harmony^[^ ^63^ ^]^ (v0.1.1) package was used to remove batch effects between two samples. Then, the first 30 principal components (PCs) was utilized to perform Uniform Manifold Approximation and Projection UMAP dimension reduction. After calculating the k‐nearest neighbors and constructing the Shared Nearest Neighbor (SNN) graph, an appropriate resolution was selected for unsupervised clustering. For the sub‐clustering of cells, after filtering out the major cell types, the aforementioned workflow was followed for integration and clustering.

### Annotation of scRNA Data

The Find All Markers function was used to identify DEGs (differentially expressed genes) for each cluster. Genes were considered as significantly DEGs if they had a *P*‐value<0.05 and a log2FC>0.5. By setting these criteria, it was ensured that the selected genes exhibited both statistical significance (*P*‐value) and a substantial magnitude of change in expression. Then, based on the marker genes identified in the reference literature, cell annotation was performed.

### Tissue Cryosection and Section Flattening

Before cryosection, the temperature of the cooling chamber was set to −20 °C. The tools involved (chip, forceps, brush, and blade) were placed in the cryostat chamber in advance for pre‐cooling. Immediately before sectioning, anterior and posterior blocks were placed into two separate cryostats (Thermo Fisher Cryostar NX50). At each desired coronal coordinate, cryosection was performed to obtain one 10‐µm section for Stereo‐seq, two 10‐µm sections for immunohistochemical staining, and three to five 50‐µm sections for scRNA‐seq. Between successive days of sectioning, the tissue blocks were stored at −80 °C. The thin Stereo‐seq sections were first flattened on a cold metal plane (−20 °C) in a cryostat with a soft brush and plastic tweezers. Then the section was carefully placed onto the precooled Stereo‐seq chip (−20 °C). To attach a tissue section progressively on the entire chip, a Stereo‐seq section was placed manually on the operator's hand to gradually raise the section temperature on Stereo‐seq chip. This procedure enables tissue attachment without air bubbles and tissue folding.

### Stereo‐Seq Library Preparation and Sequencing

The 10 µm frozen sections of mouse carotid artery plaques were evenly distributed onto a stereo‐seq chip (1 cm × 1 cm). The chip was placed on a warming plate at 37 °C for 3 min and fixed in methanol at −20 °C for 30 min. The chip was incubated with 100 µL 0.1% pepsin at 37 °C for 12 min for permeabilization and washed with 0.1× SSC buffer containing 0.05 U/µL RNase inhibitor. RNA captured by the DNA nanoball on the chip was reverse transcribed at 42 °C for 90 min. Tissue was removed from the chip by incubating with tissue removal buffer at 55 °C for 10 min. After washing with 0.1× SSC buffer, the chip with cDNA was incubated with 400 µL cDNA release buffer at 55 °C for 4 h cDNA was purified and amplified using cDNA primer. A total of 20 ng of cDNA was fragmented, amplified, and purified to generate each cDNA sequencing library. The cDNA library was sequenced on an MGI DNBSEQ‐Tx sequencer with the read length of 50 bp for read 1 and 100 bp for read 2. The reads obtained from sequencing were aligned to the mouse reference genome using the STAR1 aligner.

### Stereo‐Seq Data Processing and Image‐Based Single‐Cell Segmentation

The ssDNA staining image of a section was used to identify the cell region of the Stereo‐seq data generated from the same section. Gray‐scale maps of Stereo‐seq data were first generated, with each one pixel represented one DNA Nano Ball (DNB). To obtain single‐cell resolution spatial data, he ssDNA image was aligned with spatial expression data using tracking lines. A deep learning model from the stereopy^[^ ^64^ ^]^ (v1.2.0) package was then employed to identify the individual cell contours in the ssDNA image. Subsequently, the watershed algorithm^[^ ^65^ ^]^ was applied to assign DNBs within each contour to the respective cell.

### Mapping and Annotating Stereo‐Seq Dataset

The mapping and annotation of spatial data was accomplished through Seurat package. High‐quality single‐cell data was used as the reference dataset and spatial data as the query dataset. Both datasets were standardized using the SCTransform function. The FindTransferAnchors function was then used to identify anchor genes between the two datasets. Finally, the TransferData function was utilized to add categorical information from the corresponding single‐cell data to each cell in the spatial dataset. The cell type was screened with the highest probability as the annotation for this cell.

To further improve the accuracy of spatial annotation, corrections were performed using known marker genes of identified cell types. The average expression levels of cell type‐specific markers were used in scRNA‐seq as thresholds for correction. Specifically, corrections were applied for macrophages (*C1qa*, *C1qb*, *C1qc*), SMCs (*Acta2*, *Myh11*, *Tagln*), fibroblasts (*Col1a1*, *Col3a1*, *Dcn*), and T cells (*Cd3d*, *Cd3e*, *Cd3g*, *Cd4*, *Cd8a*, *Cd8b1*, *Nkg7*). This correction step allowed to refine the spatial annotation and enhance its accuracy.

### Calculation of Gene Sets Signature Score

The Add Module Score_UCell function from the Ucell^[^ ^66^ ^]^ (v2.4.0) package was utilized to score the enrichment level of gene sets. This function allowed to assess the degree of enrichment for a given gene set.

### Leucine Deprivation or Excess Supplementation In Vitro

Leucine‐deficient and leucine‐sufficient medium were homemade. Leucine‐free (Leu‐D) medium was prepared using DMEM powder (US Biological Life Science, D9816) or RPMI 1640 powder (US Biological Life Science, R8999‐03) and Glutamine (the same concentration as commercially available DMEM medium or RPMI 1640 medium, GIBCO, 35050‐061) and supplemented with 10% FBS. Leucine‐sufficient (Leu‐S) medium was prepared using commercially available DMEM medium or RPMI 1640 medium supplemented with L‐Leucine (10 mM, sigma, L8912). These media were adjusted to pH 7.5, filter‐sterilized (0.2 mM), and supplemented with 10% dialyzed FBS before use.

### RNA Extraction, Reverse Transcription, and Quantitative RT‐PCR

Total RNA from cultured macrophages or tissue was extracted using TRIzol reagent (Invitrogen, Carlsbad, CA, USA) and reverse‐transcribed using Transcriptor First Strand cDNA Synthesis Kit (Roche Diagnostics, Risch‐Rotkreuz, Switzerland) following the manufacturer's protocol. Quantitative real‐time PCR was performed using Fast Start Universal SYBR Green Master Mix (Roche Diagnostics). The mRNA expression levels detected in each sample were normalized to β‐actin levels. Results were analyzed with the ΔΔCt method. The primers are listed in Key Resource Table.

### Western Blot

For western blot analysis of protein levels, protein extracts from cultured macrophages and tissue were obtained using ice‐cold RIPA lysis buffer supplemented with protease and phosphatase inhibitors (Roche Applied Science). The protein concentration was quantified by BCA Protein Assay kit (Beyotime). Equal amounts of protein lysates were resolved by SDS‐PAGE, and primary antibodies were used for immunoblotting. An imaging system with Tanon 5100 was used for image acquisition, and Fiji ImageJ software was used for band densitometric analysis. Protein levels were normalized to internal control proteins.

### Confocal Microscopy and Staining of Mitochondria or Lipid Droplet

RAW264.7 Cells, THP‐1 cells or BMDMs were seeded at 3‐5x10^4^ cells/well on glass coverslips in 48‐well dishes. After treatment, for staining of mitochondria, cells were stained with MitoTracker Deep Red (100 nM) at 37 °C for 30 min, washed twice with pre‐warmed PBS, and fixed for 15 min in pre‐warmed 4% PFA. For staining of lipid droplet, treated cells or tissue slices were stained with BODIPY^493/503^ (1 µM for cells, 100 µM for tissue slices) at 37 °C for 5–10 min, washed twice with pre‐warmed PBS, and fixed for 15 min in pre‐warmed 4% PFA. The coverslips were mounted onto glass slides with antifade reagent and DAPI to stain nuclei. Fluorescence images were acquired using Zeiss LSM 800 laser‐scanning confocal microscope. All images from the same experiment were processed in the same way using the same parameters. The excitation wavelengths for MitoTracker Deep Red and DAPI were 644 and 405 nm, respectively. Quantitative analysis of fluorescence intensity was performed using ZEN 3. Quantitative analysis of fluorescence co‐localization was performed with Fiji ImageJ.^[^ ^67^, ^68^, ^69^ ^]^

### Flow Cytometry

Cells were lifted from culture plates with PBS. Then cells were pelleted by centrifugation at 1000 rcf for 3 min and were resuspended in PBS with 2% FBS. For blood cells and BMDMs, cells were stained with live/dead fixable dye (BioLegend) for 10 min at 4 °C, washed in flow buffer and then stained. For intracellular staining (CCR2), cells were fixed and permeabilized using Intracellular Fixation & Permeabilization Buffer Set (Thermo Fisher). For secretory proteins (CD5L), cells were pretreated with Cell Stimulation Cocktail (Thermo Fisher) for 6 h to block secretion and allow intracellular accumulation. For mitochondria staining, cells were stained with MitoTracker Deep Red FM (100 nM, Thermo Fisher) at 37 °C for 30 min. For detection of lipids, cells were stained with BODIPY^493/503^ dye (1 µM, MCE) at 37 °C for 30 min. For detection of mitochondrial membrane potential, cells were stained with JC‐1 working solution at 37 °C for 20 min, using CCCP (10 mM)‐treated for 20 min cells as positive control. After washing, flow cytometric analysis was performed (FACSCanto II, BD Biosciences). JC‐1 exhibits distinct fluorescence properties depending on mitochondrial membrane potential: in healthy mitochondria with normal membrane potential, JC‐1 forms aggregates within the mitochondrial matrix that emit strong red fluorescence (Ex = 488 nm, Em = 595 nm, PE channel). When mitochondrial membrane potential decreases, JC‐1 exists as monomers that produce green fluorescence (Ex = 488 nm, Em = 530 nm, FITC channel). Unstained cells were used as negative controls for background correction. The data were analyzed by FlowJo v.10 software.

### Transmission Electron Microscopy (TEM)

Cells were fixed in 2.5% glutaraldehyde solution (Sigma) (prepared in PBS, 0.2 M and pH 7.4) for 4 h at 4 °C and then post‐fixed with 1% osmium tetroxide solution (Electron Microscopy Sciences) (prepared in PBS, 0.2 M and pH 7.4) for 1 h at room temperature. Subsequently, the samples were washed three times with 0.1 M PBS (PH7.4) for 15 min each time, dehydrated through a graded series of ethanol to acetone, infiltrated, and embedded in epoxy resin (Sigma) that was polymerized in 60 °C thermostats for 48‐h. The cell blocks were cut into ultrathin sections (70 nm thick) that were collected on formvar‐coated nickel slot grids and stained with 2% uranyl acetate (Electron Microscopy Sciences) and 0.4% lead citrate (Sigma) for 15 min at room temperature. The ultrathin sections were examined using transmission electron microscope (TEM, Tecnai G2 Spirit BioTWIN).

### Seahorse Metabolic Assays

OCR and ECAR were analyzed using an XF96 Extracellular Flux Analyzer (Seahorse Biosciences) according to the manufacturer's instructions. Macrophages were seeded at 4×10^4^ cells/well. Cells were cultured for the indicated time, followed by incubating with the assay medium. Mitochondrial oxygen consumption was measured under basal conditions throughout treatment with the addition of oligomycin (1.5 µM), FCCP (1.5 µM), and rotenone/antimycin A (0.5 µM) to assess maximal mitochondrial respiration. Extracellular acidification rate was measured under basal conditions throughout treatment with the addition of glucose (10 mM), oligomycin (1 µM), and 2‐Deoxy‐DGlucose (50 mM) to assess Gly capacity.

### Cell Migration Assay

Blood monocytes (2×10^5^) were seeded in the Transwell inserts with 100 µL of serum‐free DMEM. Both upper and lower chambers contained antibiotics (100 U/mL penicillin, 0.1 mgmL^−1^ streptomycin) and 20% L‑929‐conditioned medium. The lower chamber contained 600 µL DMEM with 10% FBS and 10 ngmL^−1^ CCL2 (Peprotech). After 12 h, migrated cells were counted.

### Glucose Uptake Assay

Cells (1×10^6^) were incubated in pre‑warmed glucose‑free medium for 15 min at 37 °C, followed by a 15‑min incubation with pre‑warmed Probe solution (Dojindo) under the same conditions. Glucose uptake was assessed by flow cytometry after washed twice with pre‐chilled WI Solution (1×).

### Mitochondrial Fractionation

Mitochondrial fractionation was performed using Mitochondria Isolation Kit (Abbkine, Cat# KTP4003) according to the manufacturer's instructions. Briefly, 1×10^6^‐1×10^7^ cells were collected and incubated in an ice‐cold mitochondria isolation buffer for 15 min. After centrifugation at 600 x *g* for 10 min. nuclei and unbroken cells were discarded, and the supernatant enriched in the cytosolic fraction was obtained. Following, the supernatant was placed into a fresh tube and centrifuged to enrich the mitochondrial fraction at 3000 x *g* for 15 min at 4 °C. Mitochondria were pelleted and washed with storage buffer. One‐third of the mitochondrial fraction was pelleted as “untreated mitochondrial fraction”. Two‐thirds of the mitochondria were digested with 25 µgml^−1^ proteinase K (Thermo Fisher, Cat# 25 530 049); half of them were treated with 1 mM PMSF followed by washing twice with storage buffer containing PMSF, and the other half was treated with 1% Triton‐X‐100. The total cell and mitochondrial fractions were lysed in the lysis buffer containing protease inhibitor cocktail (Roche, Cat# 4 693 116 001) before loading on the SDS–PAGE.

### Co‐Immunoprecipitation

Protein collection and quantification were performed sequentially as described above. The cell lysate was incubated with primary antibody overnight at 4 °C. Irrelevant mouse (Sigma, Cat# 12‐371) or Rabbit IgGs (CST, Cat#2729) were used as negative control. The following day, protein A/G magnetic beads (Bimake, Cat# B23201) were subsequently added and incubated for 6 h with rotation at 4 °C. And then washed three times with immunoprecipitation washing buffer. The immunocomplexes were eluted with 50 µl SDS‐PAGE sample buffer (Beyotime), boiled for 5 min, and then detected by western blotting.

### Proximity Ligation Assay (PLA)

The PLA was performed following the DUOLINK PLA fluorescence protocol (Sigma). After treatment, cells plated on glass coverslips were washed with PBS three times, then fixed with 4% paraformaldehyde (PFA) and permeabilized using 0.1% Triton X‐100 (Sigma–Aldrich) in PBS. After incubation of primary antibodies in the DUOLINK Antibody Diluent at 4 °C overnight. Duolink In Situ PLA Probe Anti‐Rabbit PLUS and Anti‐Mouse MINUS were added and incubated for 2 h at 37 °C. Finally, the coverslips were mounted onto glass slides with DAPI to stain nuclei. Images were acquired by a Zeiss LSM 980 laser‐scanning confocal microscope and analyzed using the ImageJ software (version 2.10). The number of PLA signals per cell was quantified from the maximal intensity projection of each image.

### Molecular Dynamics Simulations

SLC7A5 (residue 51‐527) and PGAM5 (residues 2‐36) structures were extracted from PDB 6IRS and PDB 7QAM, respectively. PGAM5 was first aligned with 4F2hs's transmembrane helix in PDB 6IRS using Pymol, and was then copied and pasted into SLC7A5's PDB file, thus constructing the model 1 SLC7A5‐PGAM5 complex. The PGAM5 in model 1 was rotated 180 degrees along the Z‐axis to account for the alternative transmembrane helix packing, and this new complex was termed model 2. After preparing PDB files of the SLC7A5‐PGAM5 complex, the input files for molecular dynamics (MD) simulations were generated via CHARMM‐GUI webserver, with the complex inserted into a 120–120 °A square POPC membrane. The Charmm36m force field parameters were used to describe the system, and hydrogen mass repartitioning was conducted to enable a 4 fs time step in the production stage. The system was first energy‐minimized with 10.0 kcal/mol/°A2 harmonic constraints on protein, and 2.5 kcal/mol/°A2 on POPC. After minimization, six cycles of equilibration with each lasting 100 to 500 ps at 300 K were performed. The harmonic constraints were gradually diminished to zero as cycle number increases, with the last cycle having constraints completely removed. Time‐step was 1 fs for the first three cycles and 2 fs for the remaining cycles. After equilibration, a 500 ns‐long production run was conducted in NPT ensemble at 300 K using a 4 fs time step and 12 °A non‐bonded interaction cutoff. MD simulations were performed using the Amber20 package.

### Statistical Analysis

Data were analyzed by GraphPad Prism 9.0 Software and presented as the means ± SEM. Group sizes commonly applied in murine studies were used. Sample numbers of data obtained for physiological parameters or on mouse tissue refer to the number of individual mice entering the analysis as specified in the figure legends. Unpaired 2‐tailed Student's *t*‐test or one‐way ANOVA followed by Tukey post‐test was applied for the comparison of normally distributed data. A comparison of two groups with matched pairs of samples was performed with a paired two‐tailed Student's *t*‐test. For non‐normally distributed data, Mann‐Whitney *U* tests or Kruskal‐Wallis test with Dunn's multiple comparisons correction were performed. For statistical analyses of groups comparing two variables (e.g., genotype and treatment), a two‐way ANOVA was conducted, followed by Tukey's post hoc multiple comparison test. All data were analyzed using two‐tailed tests unless otherwise specified, and *P*‐value of less than 0.05 was considered statistically significant. For animal studies, the sample size for experiments was based on power calculations and our experience (s.d. of atherosclerotic lesion ≈5%, α = 0.05, power = 20% and detecting differences of 10%–20% in lesion area between two groups, *n* = 4‐10 mice were required). All atherosclerosis studies in this study exceeded the minimum number of mice needed for these sample size calculations and were denoted in the figures or legends. Mice in the study were randomly allocated to experimental groups. The investigators were blinded to group allocation during experiments and outcome assessment whenever possible. The representative image was selected from a repeated experiment that best matched the mean value.

### Ethical Approval

All applicable institutional and/or national guidelines for the care and use of animals were followed. The Research Ethics Committee of the Second Affiliated Hospital of Harbin Medical University (YJSKY2022‐516 and YJSDW2022‐159)

## Conflict of Interest

The authors declare no conflict of interest.

## Author Contributions

S. Z., X. W., and Q. L. contributed equally to this work. J.‐W.T. conceived the project. S.Z. executed most of the experiments under the supervision of J.Z., S.‐J.L., and J.‐W.T.; X.‐Y.W. helped with most of the experiments. B.S. performed the protein interaction analysis. Q.‐S.L., with the assistance of X.‐W.X., G.X., and J.X. performed most of the bioinformatics analysis. W.‐J.N. and S.‐Q.W. performed parts of the flow cytometry, western blot, and histopathology experiments. C.J., P.Z., F.‐Y.H. Y.‐D.W. performed the human cohort analysis. X.P., G.‐Y.Z., and X.‐W.X. helped with genotype identification. X.G. helped with part of the animal experiment. S.Z., X.‐Y.W., analyzed data. S.L. provided human samples. S.Z., J.Z., S.‐J.L., and J.‐W.T. wrote the paper.

## Supporting information



Supporting Information

## Data Availability

The data underlying this article will be shared on reasonable request to the corresponding author. The Stereo‐seq data could be obtained from the CNGB Spatial Transcript Omics DataBase (https://db.cngb.org/stomics/) with an accession number STT0000095. The single‐cell sequencing data could be obtained from the CNGB Sequence Archive (CNSA) of China National GeneBank DataBase (CNGBdb) (https://db.cngb.org/cnsa) with an accession number CNP0005765. The processed data and analysis codes are available upon request from the corresponding author.
