# Supplementary material for: Leucine‐Dependent SLC7A5–PGAM5 Interaction Promotes Advanced Atherosclerosis Through Hindering Mitochondrial Function of Macrophages
Source: Adv Sci (Weinh). 2025 Nov 21;13(7):e18359. doi: 10.1002/advs.202518359 (PMC12866778; doi:10.1002/advs.202518359)
Supplement: Supplementary file 1 — Supporting Information [file ADVS-13-e18359-s001.docx]

**Leucine-Dependent SLC7A5–PGAM5 Interaction Promotes Advanced Atherosclerosis through Hindering Mitochondrial Function of Macrophages**

Shan Zhong[^1^](#二院心内)^,^[^3^](#泛血管)^,^[^4^](#心肌缺血)^#^, Xueyu Wang[^1^](#二院心内)^,^[^3^](#泛血管)^,^[^4^](#心肌缺血)^#^, Qingsong Li[^5^](#王岳1)^,^[^6^](#王岳2)^,#^, Siqi Wang[^1^](#二院心内)^,^[^3^](#泛血管)^,^[^4^](#心肌缺血), Bin Sun[^7^](#孙斌), Wenjun Ni[^3^](#泛血管)^,^[^7^](#孙斌)^,^[^8^](#药学院), Gengyu Zhou[^1^](#二院心内)^,^[^3^](#泛血管)^,^[^4^](#心肌缺血), Fan Wang[^1^](#二院心内)^,^[^3^](#泛血管)^,^[^4^](#心肌缺血), Xianwei Xie[^1^](#二院心内)^,^[^3^](#泛血管)^,^[^4^](#心肌缺血), Cheng Jin[^3^](#泛血管), Gang Xu[^9^](#生物信息学院), Peng Zhao[^1^](#二院心内)^,^[^3^](#泛血管)^,^[^4^](#心肌缺血), Xiang Peng[^1^](#二院心内)^,^[^3^](#泛血管)^,^[^4^](#心肌缺血), Feiyuan Han[^1^](#二院心内)^,^[^3^](#泛血管), Xiangwen Xi[^3^](#泛血管)^,^[^10^](#祥文海南), Yidan Wang[^1^](#二院心内)^,^[^3^](#泛血管), Juan Xu[^9^](#生物信息学院), Yue Wang[^5^](#王岳1)^,^[^6^](#王岳2)^,^[^11^](#王岳3), Xia Gu[^3^](#泛血管)^,^[^4^](#二院心内)^,^[^12^](#顾霞影像中心), Shuo Li[^1^](#二院心内)^,^[^3^](#泛血管), Jian Zhang[^13^](#张健)*, Shuijie Li[^8^](#药学院)^,^[^14^](#刘惠彬药学部)^,^[^15^](#李水洁药学院实验室)*, Jinwei Tian[^1^](#二院心内)^,^[^2^](#寒冷全重)^,^[^3^](#泛血管)^,^[^4^](#心肌缺血)^,^[^16^](#二院老年病)^,^[^17^](#主要通讯)*

**Author affiliations**

1. Department of Cardiology, The Second Affiliated Hospital of Harbin Medical University, Harbin, China.

2. State Key Laboratory of Frigid Zone Cardiovascular Diseases (SKLFZCD), Harbin Medical University, Harbin, China.

3. Heilongjiang Provincial Key Laboratory of Panvascular Disease, Harbin, China.

4. The Key Laboratory of Myocardial Ischemia, Ministry of Education, Harbin Medical University, Harbin, China.

5. BGI Research, Beijing, China

6. Collaborative Innovation Center for Molecular Imaging of Precision Medicine, Shanxi Medical University, Taiyuan, Shanxi, People's Republic of China

7. Research Center for Pharmacoinformatics (the State-Province Key Laboratories of Biomedicine-Pharmaceutics of China), College of Pharmacy, Harbin Medical University, Harbin, China

8. College of Pharmacy, Harbin Medical University, Harbin, China

9. College of Bioinformatics Science and Technology, Harbin Medical University, Harbin, Heilongjiang

10. Department of Cardiology, the Second Affiliated Hospital of Hainan Medical University, Haikou, China.

11. State Key Laboratory of Quality Research in Chinese Medicine and Institute of Chinese Medical Sciences, University of Macau, Macao, China

12. Cardiovascular Imaging Center, The Second Affiliated Hospital of Harbin Medical University, Harbin, Heilongjiang, China

13. School of Pharmacy, East China University of Science and Technology, Shanghai, China.

14. Department of Biopharmaceutical Sciences, College of Pharmacy, Harbin Medical University, Harbin, China.

15. Heilongjiang Province Key Laboratory of Research on Molecular Targeted Anti-Tumor Drugs

16. Department of Geriatrics, The Second Affiliated Hospital of Harbin Medical University, Harbin, China.

17. Lead contact

^#^ The authors have contributed equally to this work.

*Corresponding authors:

Jinwei Tian, MD, PhD

Department of Cardiology

The Second Affiliated Hospital of Harbin Medical University

150086 Harbin, China

Email: [tianjinweidr2009@163.com](mailto:tianjinweidr2009@163.com)

Shuijie Li, MD, PhD

College of Pharmacy

Harbin Medical University

150086 Harbin, China

Email: shuijie.li@hrbmu.edu.cn (S.-J.L.)

Jian Zhang, MD, PhD

Shanghai Key Laboratory of New Drug Design, School of Pharmacy

East China University of Science & Technology

200237 Shanghai, China

Email: [zhangjian_tina@163.com](mailto:zhangjian_tina@163.com) (J.Z.)

**Supplementary figure captions**

**
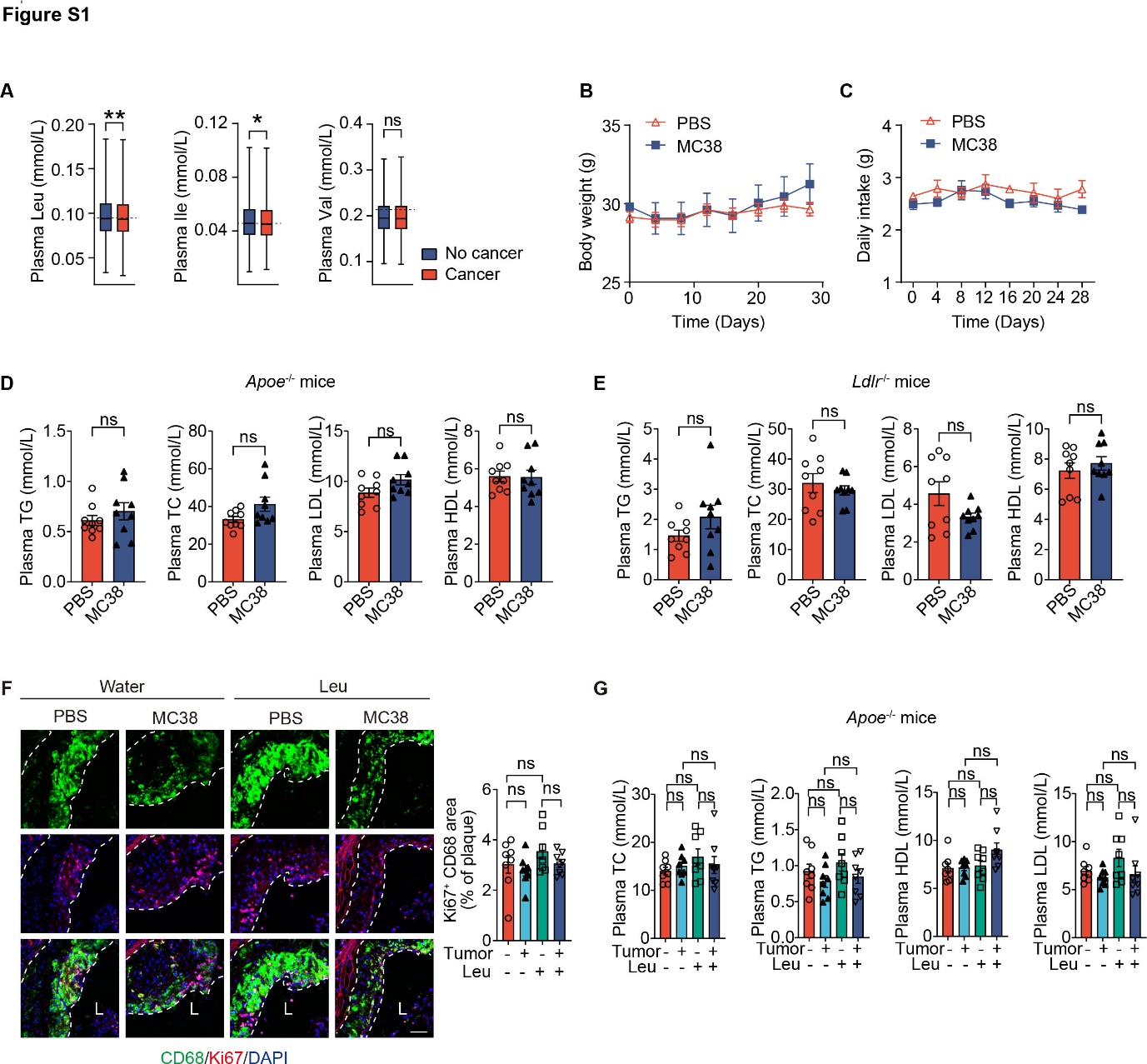
**

**Figure S1. BCAA levels in the population and body weight, food intake, and lipid levels in multiple mouse models.**

A, Plasma BCAA levels in participants from the UK Biobank (*n*=8226/group).

B-C, Body weight curve (B) and daily food intake (C) of *Apoe*^-/-^ mice that consumed a HFD for 12 weeks and then received PBS or tumor cells (*n=*9-10 mice/group).

D-E, Plasma lipid profiles in *Apoe*^-/-^ mice (D, *n=*9-10/group) and *Ldlr*^-/-^ mice (E, *n=*9-10/group) fed an HFD for 16 weeks.

F, Analysis of intra-plaque proliferation using Ki67 staining (*n=*8/group). L, lumen. Scale: 50 μm.

G, Plasma lipid profiles of *Apoe*^-/-^ mice receiving water with or without excessive leucine and treated with PBS or MC38. *n*=8 mice/group.

Data are mean±SEM. **P*<0.05, ***P*<0.01, unpaired Student’s *t*-test (A, D, E), two-way ANOVA with Dunnett correction (B, C) and one-way ANOVA with Dunnett correction (F, G).


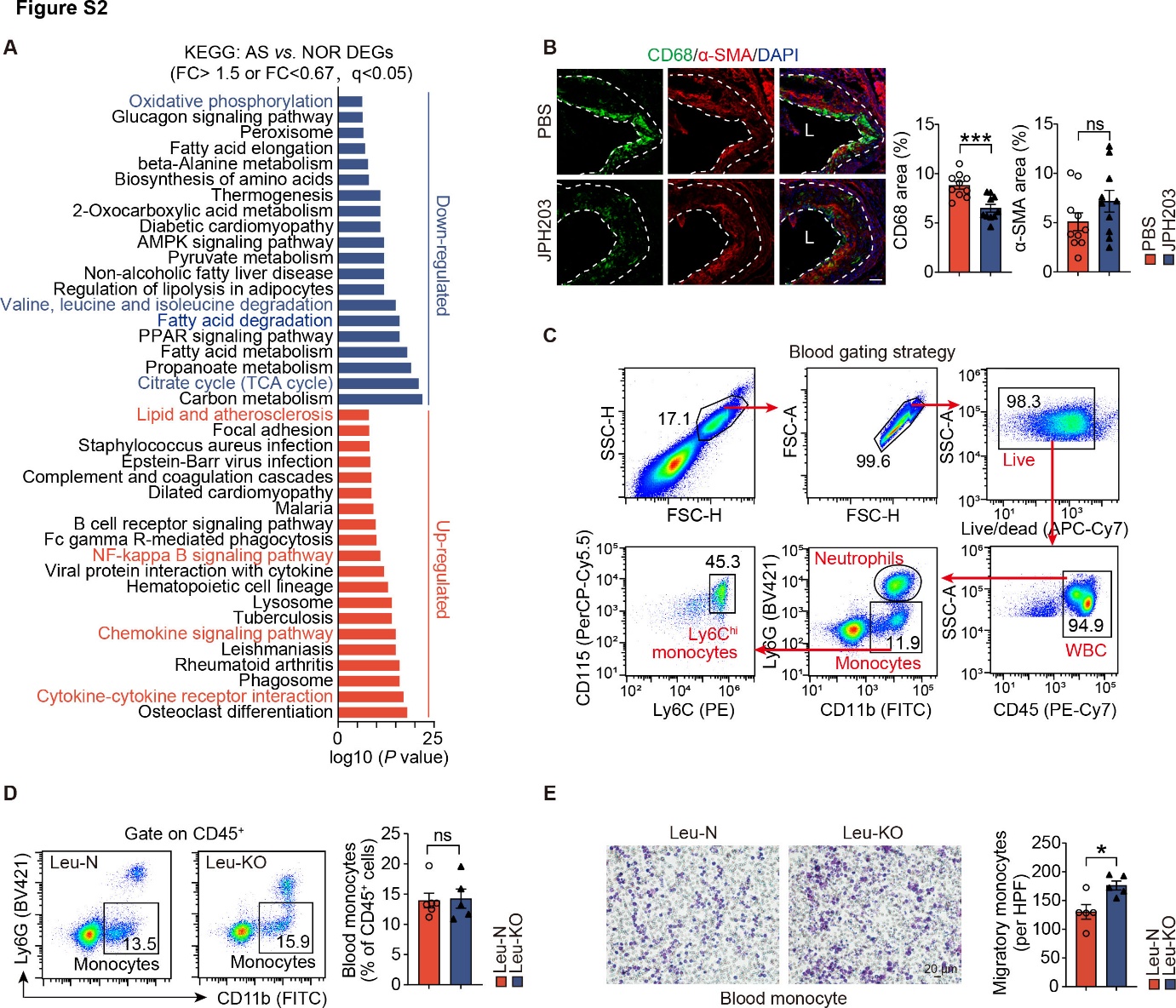


**Figure S2. Leucine restriction shapes monocyte dynamics and AS.**

A, Top 20 KEGG pathway enrichment for DEGs identified using RNA-seq of aortas from *Apoe*^-/-^ mice fed an HFD (AS) *vs.* chow-diet (NOR) for 16 weeks (FC>1.5 or FC<0.67, q<0.05) (*n=*6 mice/group).

B, Immunofluorescence staining of CD68 (macrophages) and α-SMA (smooth muscle cells) (*n=*10/group). Scale: 100 μm.

C, Gating strategy used to identify blood monocytes subsets.

D, Quantification of blood monocytes in *Apoe*^-/-^ mice were fed either a leucine-restricted high-fat diet (0% leucine; Leu-KO) or a normal high-fat diet (1.6% leucine; Leu-N) for 4 weeks. (*n=*5-6/group).

E, Transwell migration of blood monocytes isolated from indicated mice (*n=*5/group).

Data are mean±SEM. ns, not significant, **P*<0.05, ****P*<0.001, unpaired Student’s *t*-test (B, D, E).


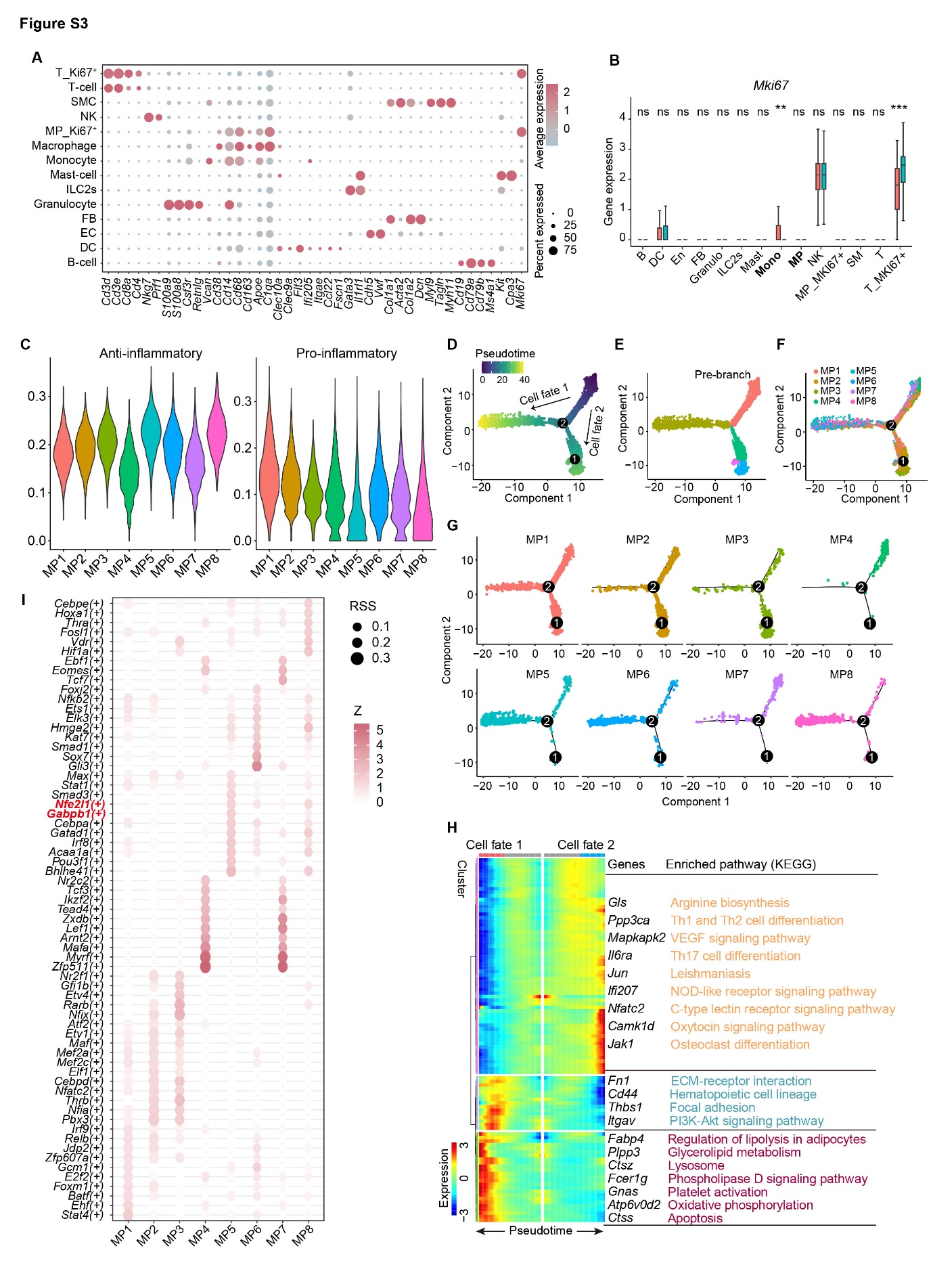


**Figure S3. Single-cell RNA seq analysis of mouse aortas.**

A, Bubble diagram showing scaled expression of marker genes for cell clusters in whole mouse aortas from *Apoe*^-/-^ mice fed an HFD for 16 weeks.

B, Single-cell analysis of the expression of *Mki67* in each cell cluster.

C, Anti-inflammatory and pro-inflammatory score of each macrophage subcluster.

D-F, The pseudotime (D) and developmental trajectory of macrophage, coloured-coded by the associated cell fates (E) and cell subclusters (F).

G, Macrophages on the pseudotime tree are color-coded by subclusters.

H, Heatmap of relative expression of differentially expressed genes between fate 1 and fate 2 cells (left) and corresponding representative gene ontology (biological process) terms (*P* adj, <0.05) of fate 1 and fate 2 cells (right).

I, PySCENIC-based inferred potential transcription factors (TFs) and regulon specificity score (RSS) for each macrophage subcluster.

Statistical significance was assessed by a one-sided t-test in the box plots (B), ns, not significant, ***P*<0.01, ****P*<0.001.


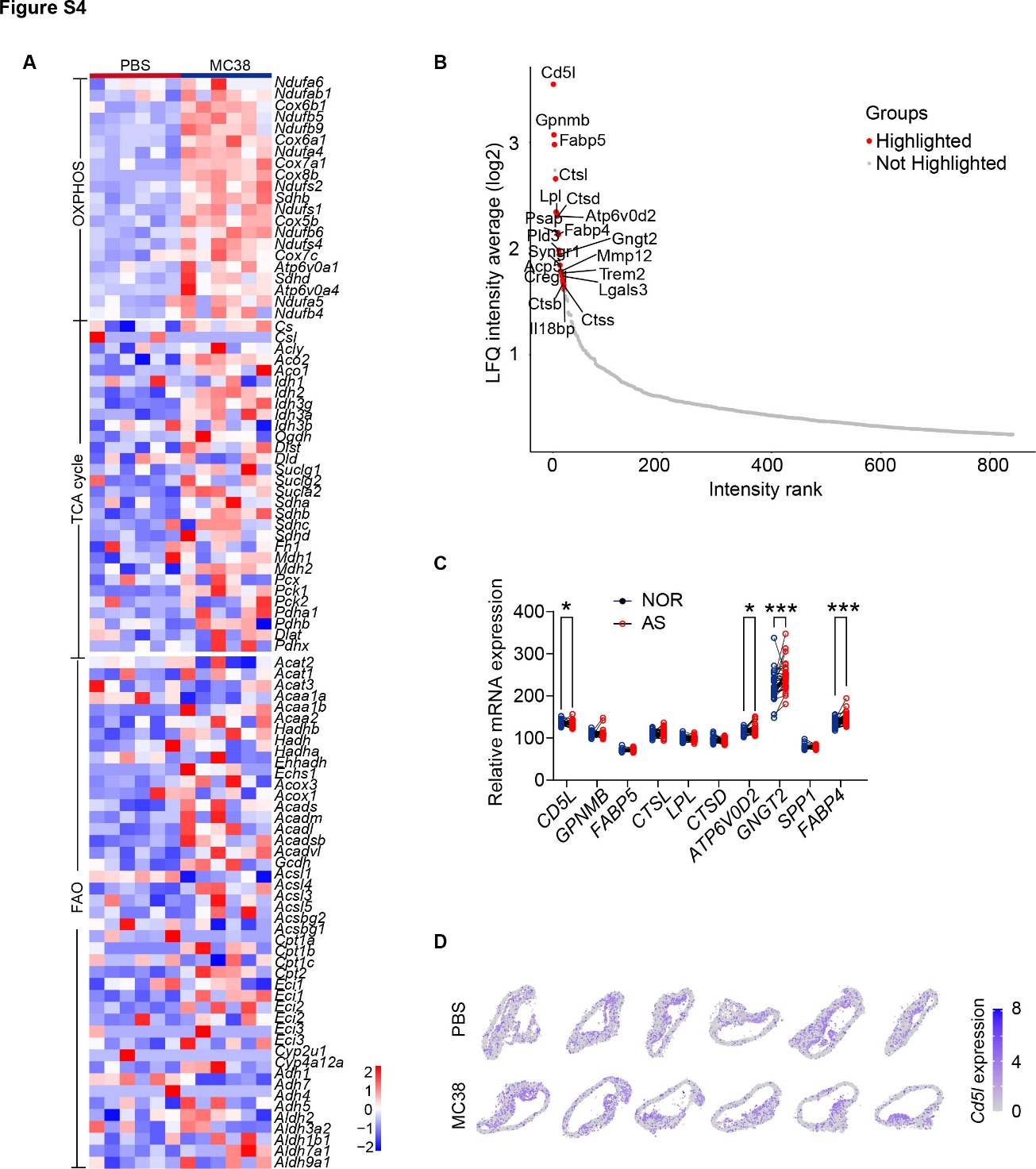


**Figure S4. Spatial transcriptome analysis of the origin of mouse ascending aortas section.**

A, Heatmap showing the expression of the three KEGG metabolic pathway (FAO, TCA cycle, and OXPHOS) gene sets from mouse ascending aorta atherosclerotic lesions from indicated groups.

B, Kurtosis range ranking map showing marker genes of *Trem2*^+^*Cd5l*^+^ macrophages (MP5) using scRNA-seq, sorted by average log2FC of gene expression.

C, MP5 marker gene mRNA levels in the human atherosclerotic arterial wall (AS) compared with paired non-atherosclerotic arterial wall (NOR) in GSE40231 dataset (*n*=40/group).

D, Spatially *Cd5l* expression patterns in aortic plaques sections from six mice in each group.

Statistical analysis was performed by paired Student’s *t*-test (C). **P*<0.05, ****P*<0.001.


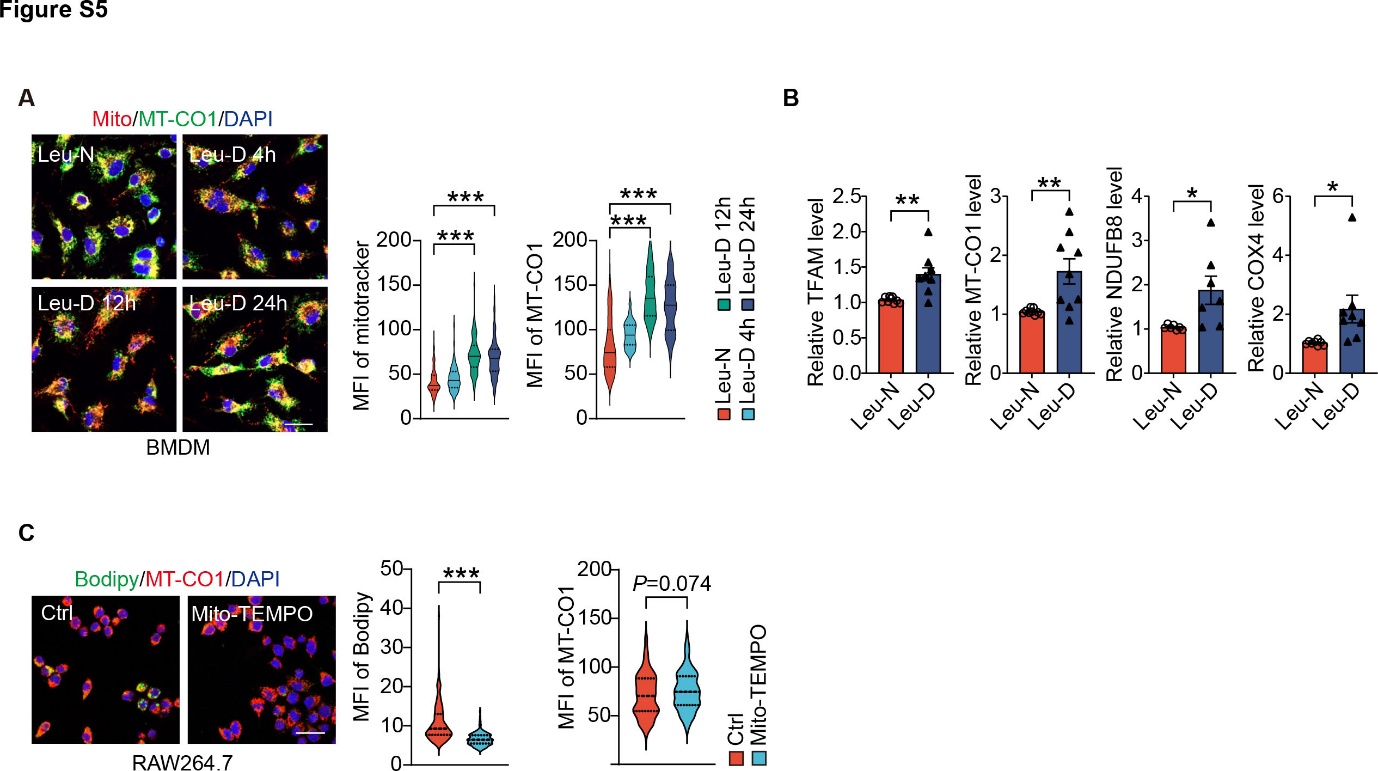


**Figure S5. Leucine deficiency enhances macrophage mitochondrial functions.**

A, Immunofluorescent staining of MitoTracker-deep red and MT-CO1 in BMDMs treated with Leu-N or Leu-D as indicated (*n*≥80 cells/group). Scale, 5 μm.

B, Relative quantification of TFAM, MT-CO1, NDUFB8 and COX4 protein in Fig. 4C (*n*≥7/group).

C, Immunofluorescent staining of BODIPY^493/503^ and MT-CO1 in RAW264.7 cells treated with ox-LDL with or without mito-TEMPO for 24 h (*n*≥100 cells/group). Scale: 20 μm.

Data are mean± SEM. **P*<0.05, ***P*<0.01, ****P*<0.001, Kruskal-Wallis test (A), unpaired Student’s *t*-test (B), and Mann-Whitney *U* test (C).


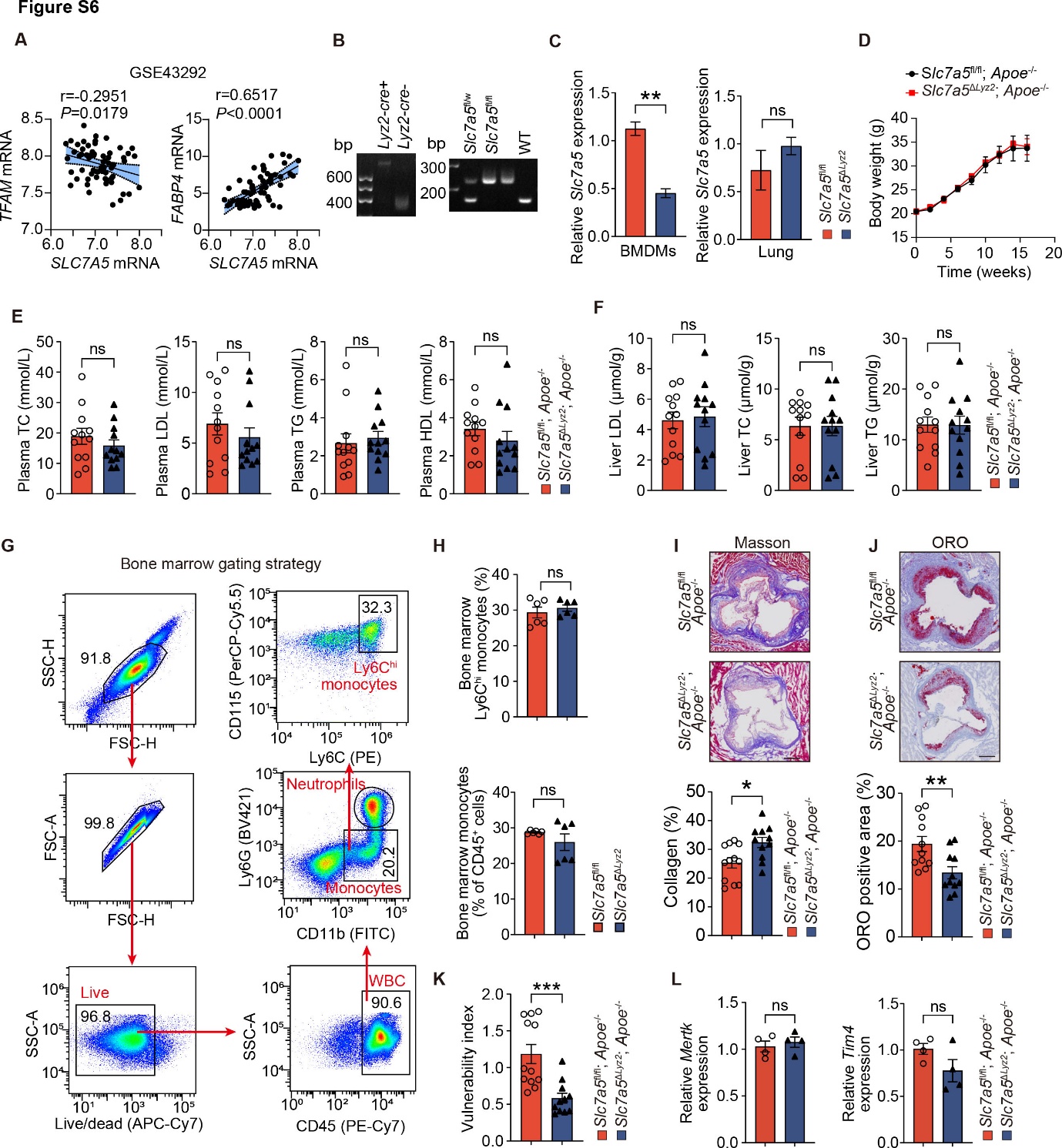


**Figure S6. Myeloid knockout of *Slc7a5* mice phenotypes.**

A, Correlation analyses of *SLC7A5* and *TFAM* (left) or *FABP4* (left) mRNA levels in human carotid atherosclerotic plaques (GSE43292; *n*=64).

B, PCR analysis of WT (Right, 4) and targeted (Right, 2-3) allele of Slc7a5 exhibited 157- and 224-bp bands, and the mutant gene of Lyz2-cre (Left, 1) exhibited 700-bp bands.

C, *Slc7a5* mRNA level in BMDMs (left) and lung tissue (right) from *Slc7a5^ΔLyz2^*; *Apoe*^-/-^ mice and *Slc7a5*^fl/fl^ mice (*n*=3 mice/group).

D-F, Changes in body weight (D, *n*=6), serum (E, *n*=12), and liver lipid (F, *n*=12) of *Slc7a5^ΔLyz2^*; *Apoe*^-/-^ and *Slc7a5*^fl/fl^; *Apoe*^-/-^ mice fed an HFD for 16 weeks.

G, Gating strategy used to identify bone marrow monocytes subsets.

H, Quantification of bone monocytes from *Slc7a5^ΔLyz2^* and *Slc7a5*^fl/fl^ mice (*n*=6 mice/group).

I-K, Masson’s (I) and ORO (J) of aortic sinus and plaque vulnerability index (K) of indicated mice (*n*=11-12 mice/group). Scale: 250 μm (high magnification).

L, Quantitative RT-PCR analysis of efferocytosis-related genes (*Mertk* and *Tim4*) in peritoneal macrophages from mice in Fig 5E (*n*=4 mice/group).

Data are mean±SEM. ns, not significant, **P*<0.05, ***P*<0.01, ****P*<0.001, Pearson’s correlation test (A), unpaired Student’s *t*-test (C, E, F, and H-L) and two-way ANOVA (D).


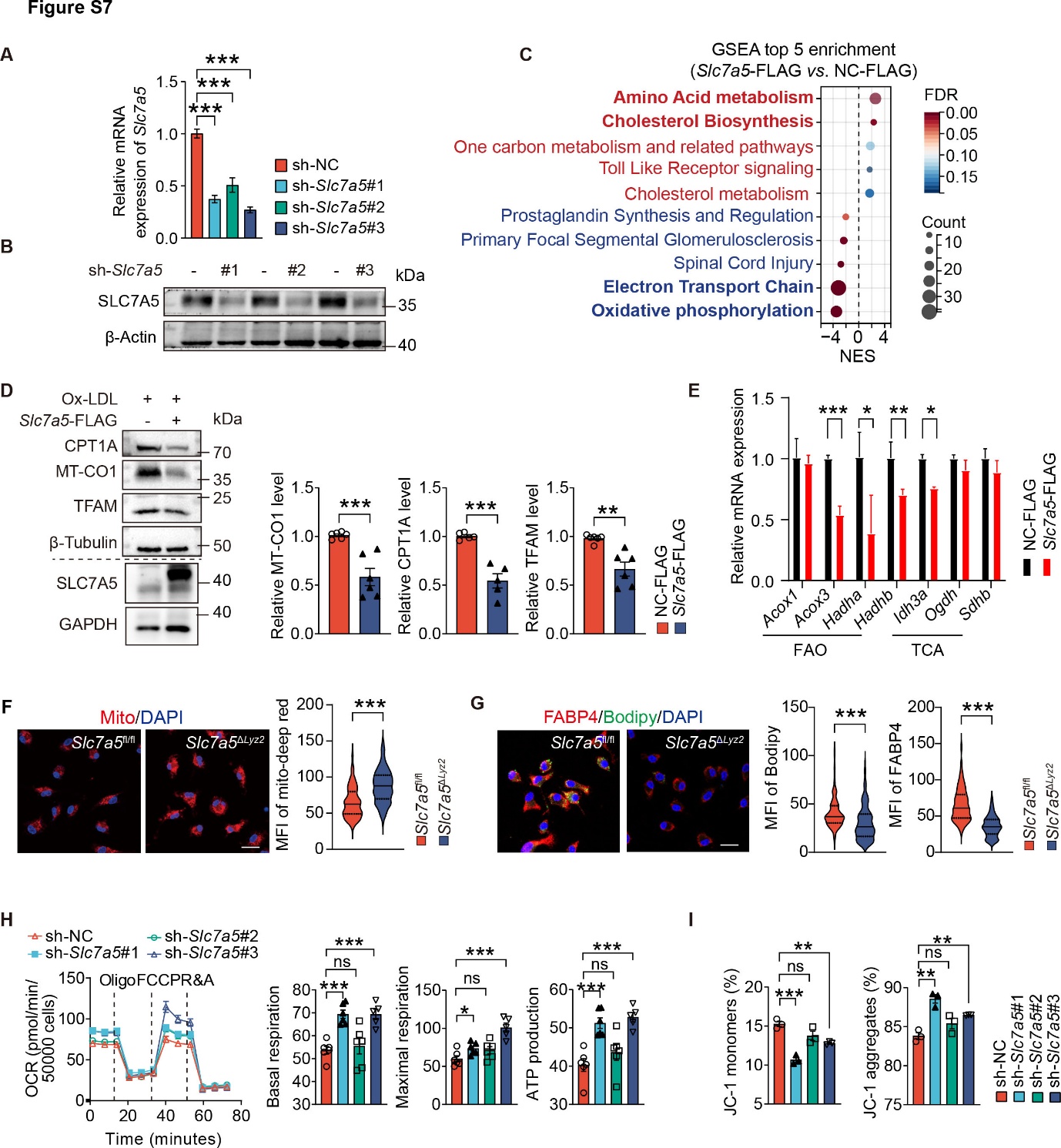


**Figure S7. *Slc7a5* knockdown or overexpression induces changes in metabolic pathways in macrophages.**

A, RT-PCR analysis of *Slc7a5* mRNA levels in RAW264.7 cells treated with or without three lines of sh-*Slc7a5*. *n*=3.

B, Immunoblot analysis of SLC7A5 in RAW264.7 cells treated with or without three lines of sh-*Slc7a5*.

C, Top 5 enriched KEGG pathways using gene set enrichment analysis (GSEA) of RAW264.7 cells (*Slc7a5*-FLAG *vs.* NC-FLAG). NES, normalized enrichment score.

D, Immunoblot analysis of the FAO- and TCA-related proteins from RAW264.7 cells after indicated treatments. *n*≥5/group.

E, Relative mRNA levels of genes encoding rate-limiting enzymes in the FAO and TCA cycle pathways in RAW264.7 cells with or without SLC7A5 overexpressing (*Slc7a5*-FLAG and NC-FLAG). *n*=3.

F, Immunofluorescence staining of MitoTracker-deep red of BMDMs from the *Slc7a5*^fl/fl^ mice and *Slc7a5^ΔLyz2^* mice following 24-h ox-LDL (50 ug/mL) treatment (*n*>100 cells from 3 mice per group). Scale: 20 μm.

G, Immunofluorescent staining of FABP4 and BODIPY^493/503^ in indicated BMDMs (*n*≥100 cells/group). Scale: 20 μm.

H, OCR of RAW264.7 cells transfected with sh-*Slc7a5* *vs*. negative control sh-NC treated with ox-LDL for 24-h (50 μg/mL) (*n*=5-6/group).

I, JC‑1 flow cytometry of mitochondrial membrane potential depolarization in indicated RAW264.7 cells (*n=*5/group).

Data are mean±SEM. ns, not significant, **P*<0.05, ***P*<0.01, ****P*<0.001, unpaired Student’s *t*-test (A, D, H and I), Mann-Whitney *U* test (F, G), and two-way ANOVA (E).


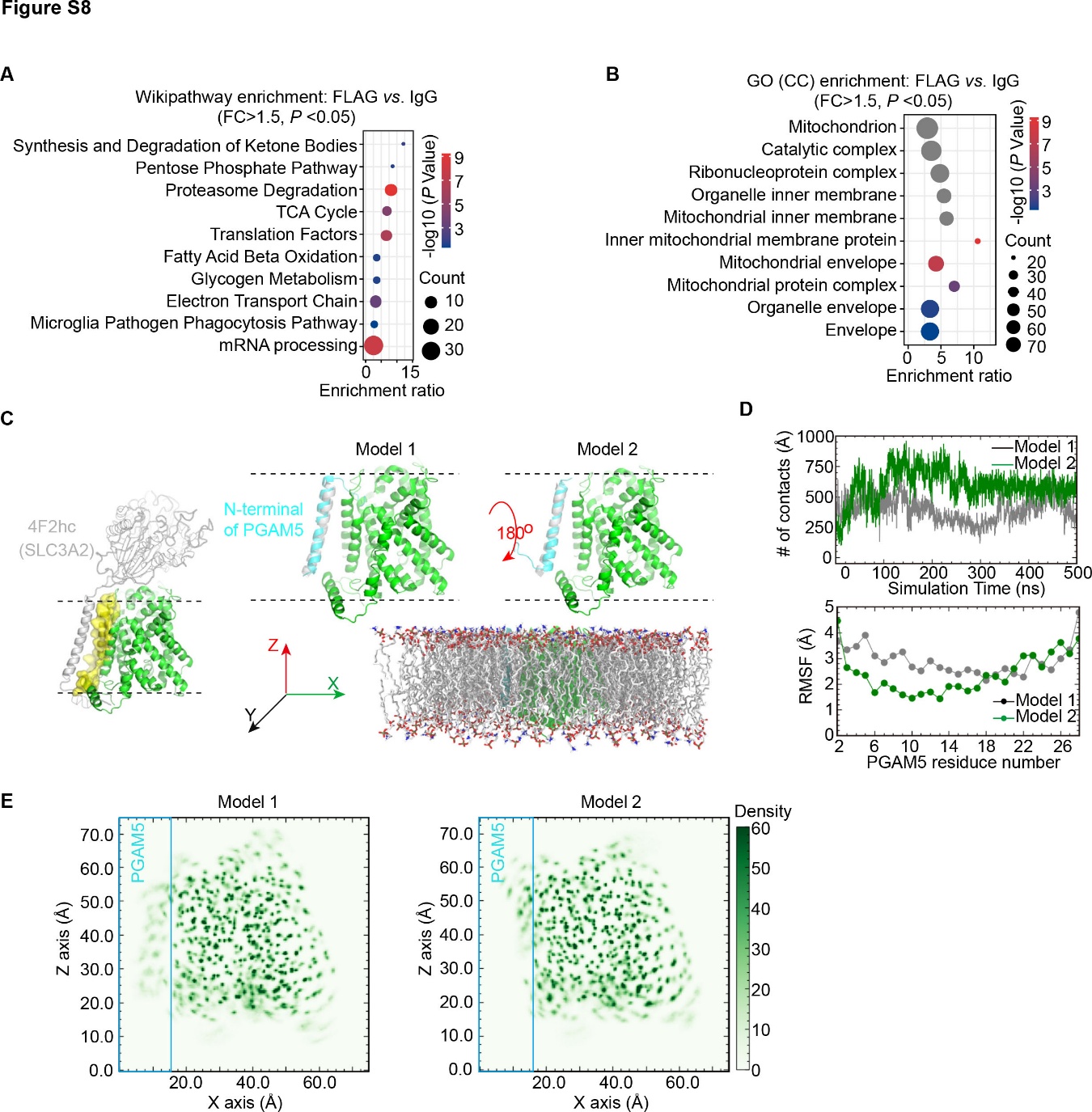


**Figure S8. PGAM5 directly binds to SLC7A5 in the mitochondria of macrophages.**

A-B, WikiPathway enrichment (A) and GO-cellular component (GO-CC) (B) analyses of proteins identified as potential SLC7A5 interactors using label-free proteomic sequencing (anti-FLAG vs anti-IgG, *P*-adj.< 0.05).

C, Reported SLC7A5-4F2hc complex structure (left). Two models of SLC7A5-PGAM5 complex in which PGAM5’s transmembrane helix (TM) was aligned onto 4F2hc’s TM and was placed adjacent to SLC7A5 are shown.

D, Number of contacts between PGAM5 and SLC7A5 during the molecular dynamics (MD) simulations. The atomic fluctuations of PGAM5 were indicated using the root mean square fluctuation (RMSF) plots.

E, Lateral projection of atom density of the SLC7A5-PGAM5 complex.


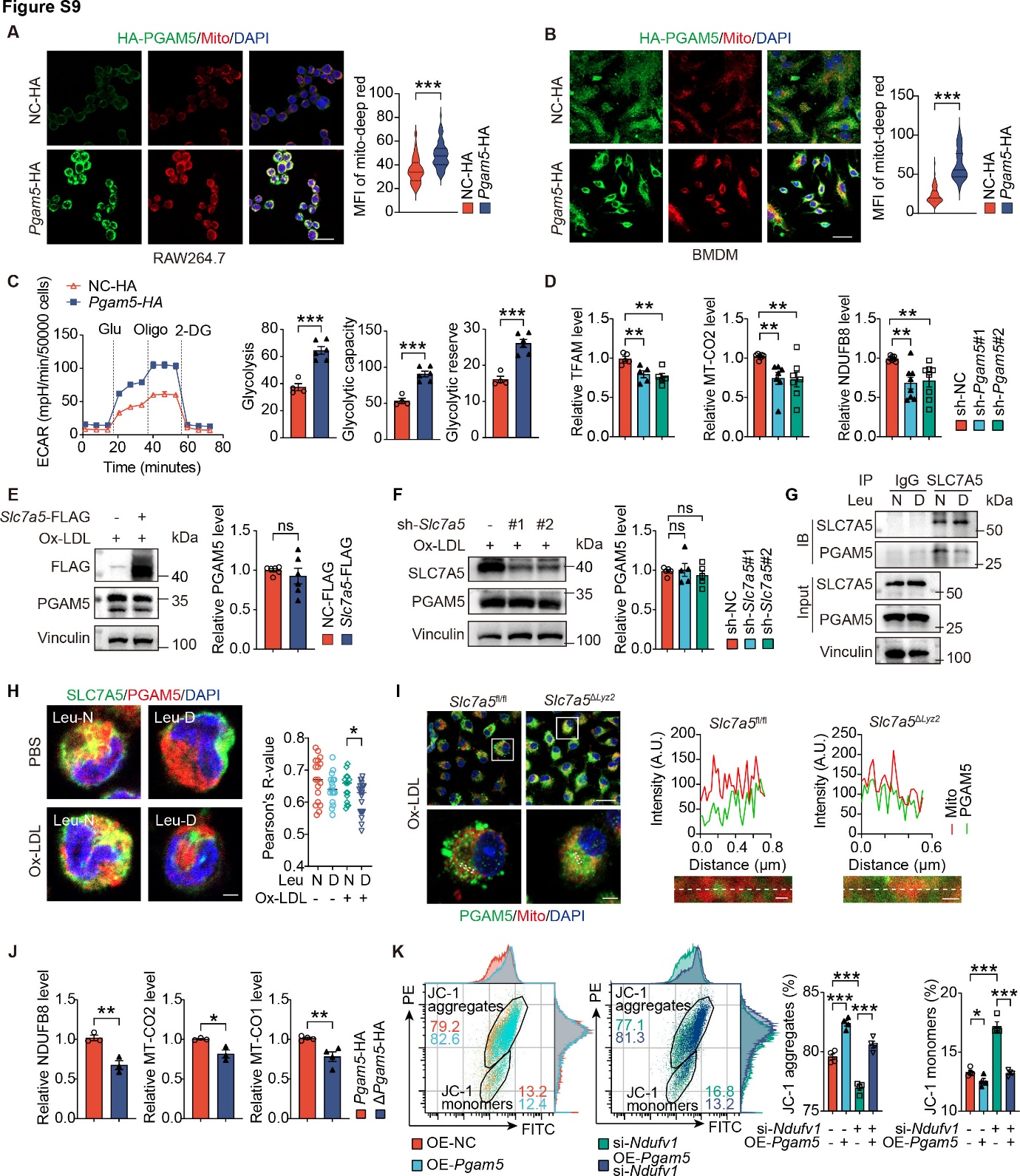


**Figure S9. Leucine deficiency impairs the PGAM5-SLC7A5 interaction, enhancing PGAM5-mediated mitochondrial function.**

A-B, Immunofluorescent staining of MitoTracker and HA (PGAM5-HA) in PGAM5-HA or NC-HA-transfected RAW264.7 cells (A, *n*≥100 cells/group) and BMDMs (B, *n*≥100 cells from 3 mice/group). Scale: 20 μm.

C, ECAR of RAW264.7 cells that were transfected with PGAM5-HA or NC-HA (*n*=7/group).

D, Relative quantification of TFAM, MT-CO2 and NDUFB8 protein in Figure 7C. *n*≥5/group.

E-F, Immunoblotting of PGAM5 in RAW264.7 cells treated with ox‑LDL (50 μg/ml) for 24 h: (E) cells transfected with NC‑FLAG or *Slc7a5*-FLAG; (F) cells transfected with sh‑NC or sh-*Slc7a5*) (*n*=5 or 6/group).

G, Co-IP of endogenous SLC7A5 and immunoblot analysis of SLC7A5 and PGAM5 in 293T cells with or without leucine deficiency.

H, Immunofluorescent staining of SLC7A5 (green) and PGAM5 (red) in macrophages derived from THP-1 cells, treated with or without ox-LDL in Leu-D and Leu-N media. Co-localization analyses are shown on the right (*n*=16-21 visual fields per group, each containing over 30 cells). Scale: 5 μm.

I, Immunofluorescent staining of MitoTracker and PGAM5 in BMDMs of the *SLC7A5*^fl/fl^ mice and *Slc7a5^ΔLyz2^* mice under 24-h ox-LDL (50 μg/mL) treatment (*n*>100 cells from 3 mice/group). Scale: 20 μm.

J, Relative quantification of NDUFB8, MT-CO2 and MT-CO1 protein in Fig 7K. *n*=3-4/group.

K, JC‑1 flow cytometry of mitochondrial membrane potential depolarization in RAW264.7 cells transfected with or without OE-*Pgam5* or si-*Ndufv1* (*n=*4/group).

Data are mean± SEM. ns, not significant, **P*<0.05, ***P*<0.01, ****P*<0.001, Mann-Whitney test (A, B) and unpaired Student’s *t*-test (C-F, H, and J-K).

**Table S1. Age and sex of participants in each group of Figure1A-1B.**

| Group | Sex (Male, %) | Age at recruitment |
| --- | --- | --- |
| Leu-0 (*n*=48972) | 10658 (21.14) | 56.17±8.22 |
| Leu-1 (*n*=48973) | 17106 (33.93) | 56.31±8.09 |
| Leu-2 (*n*=48961) | 23314 (46.25) | 56.29±8.03 |
| Leu-3 (*n*=48984) | 29046 (57.59) | 56.04±8.03 |
| Leu-4 (*n*=48975) | 31470 (62.39) | 55.87±8.01 |
| HR | 2.504 | 1.055 |
| *P*-value | <0.0001 | <0.0001 |

Data are shown as mean ± SD or frequencies (%), Cox-regression analysis. HR, Hazard Ratio.

**Table S2. Characteristics of the patients enrolled in the study of Figure S1A.**

| **Characteristics** | **No cancer**  matched (n=8226) | **Cancer**  (n=8226) | ***P* value** |
| --- | --- | --- | --- |
| Age (years) | 59.65± 6.99 | 59.65± 7.33 | 0.947 |
| Male gender (n, %) | 3123 (38.0) | 3102 (37.7) | 0.736 |
| BMI | 27.15± 4.62 | 27.21± 4.71 | 0.472 |
| Townsend | -1.41± 3.03 | -1.47± 3.03 | 0.225 |

Data are shown as mean ± SD or frequencies (%). Comparisons of continuous variables were performed by Student's *t*-test for normally-distributed variables. Comparisons of categorical variables were conducted with chi-squared test.
